# Efficacy of a Low-Purine, Energy-Restricted and Balanced Diet on Hyperuricemia and Metabolic Profiles in Gout Patients: A Randomized Controlled Trial

**DOI:** 10.3390/nu18132047

**Published:** 2026-06-23

**Authors:** Ting Zhao, Shan Li, Ruonan Wu, Liyang Zhang, Jiaxin Wen, Junqi Xiao, Duo Li

**Affiliations:** 1 Institute of Nutrition & Health, School of Public Health, Qingdao University, Qingdao 266000, China; zhaoting@qdu.edu.cn (T.Z.); lishan1@qdu.edu.cn (S.L.); ztxhhh@sina.com (L.Z.); wenjiaxin0823@163.com (J.W.); 18391648141@163.com (J.X.); 2 Chinese Center for Disease Prevention and Control, Qingdao 266000, China; 15689134965@163.com

**Keywords:** nutritional therapy, gout, obesity, serum uric acid, fractional excretion of uric acid

## Abstract

**Background/Objectives:** Nutritional therapy has emerged as a promising therapeutic strategy for the management of chronic metabolic diseases. This study aims to evaluate the efficacy of a low-purine, energy-restricted, and balanced diet (LPEB diet) in ameliorating gout conditions and improving related metabolic risk factors. **Methods:** A total of 90 patients with gout were randomly allocated to either the intervention group or the control group, with 45 cases in each group. Patients in the control group received routine basic nutritional health education. Based on the conventional education, the intervention group underwent a 42-day structured dietary intervention characterized by low purine intake, energy restriction, and balanced nutritional composition. **Results:** Compared with the control group, the intervention group showed a significant reduction in serum uric acid (sUA) level by 112.4 μmol/L (*p* = 0.007). Meanwhile, the fractional excretion of uric acid (FEUA) showed a significantly greater increase of 0.87% in the intervention group compared with the control group (*p* = 0.003), while daily purine intake was significantly reduced by 262 mg (*p* = 0.001) in the intervention group. Moreover, notable improvements in body composition were observed in the intervention group. Specifically, body mass index (BMI) decreased by 0.50 kg/m^2^ (*p* < 0.001) and visceral fat area (VFA) was reduced by 12.1 cm^2^ (*p* < 0.001), with significant intergroup differences confirmed for both indicators. **Conclusions:** This study demonstrates that an LPEB diet not only effectively reduces sUA levels by enhancing FEUA but also significantly ameliorates central adiposity and related metabolic risk factors in patients with gout.

## 1. Introduction

Gout is a common crystal deposition arthropathy caused by monosodium urate (MSU) crystal accumulation, with hyperuricemia serving as the fundamental pathological basis. Hyperuricemia develops primarily from purine metabolism disorders, impaired renal uric acid excretion, or a combination of these two pathological mechanisms. High uric acid levels are linked to inflammatory reactions and exert detrimental impacts on health [1,2]. In addition, gout patients are at high risk of severe complications, including irreversible joint destruction and progressive renal dysfunction [3]. Recurrent acute gout flares and long-term chronic complications severely impair patients’ quality of life and may lead to occupational disability, imposing a substantial socioeconomic burden [4].

Lowering serum uric acid (sUA) is the core therapeutic target for gout management. At present, pharmacotherapy remains the mainstream clinical strategy for gout treatment. Commonly prescribed urate-lowering medications are classified into two major categories: xanthine oxidase inhibitors and uricosuric agents. Although these drugs can rapidly and effectively reduce circulating sUA levels, long-term medication exposure carries potential risks of hepatorenal toxicity and gastrointestinal adverse reactions [5].

Mounting clinical evidence has shown that excessive consumption of high-purine foods is closely correlated with elevated sUA concentrations and increased frequency of acute gout flares [6,7]. Accumulated cross-sectional epidemiological data further validate that animal-based dietary patterns are positively associated with sUA levels, while plant-based dietary patterns exhibit an inverse correlation [8]. Accordingly, nutritional intervention has emerged as a crucial strategy for gout management. A low-purine diet is a cornerstone of the dietary management of hyperuricemia and gout [3]. This dietary pattern is defined by the restriction of exogenous purine intake, primarily by limiting high-purine foods such as organ meats, certain seafood, concentrated broths, and red meat, while emphasizing the consumption of low-purine foods, including fresh vegetables, fruits, whole grains, and low-fat dairy products. Despite its established physiological rationale for lowering serum urate levels, long-term adherence to a strict low-purine diet remains a significant clinical challenge. This difficulty stems from its restrictive nature, which can adversely affect patients’ social quality of life and dietary satisfaction.

The Dietary Approaches to Stop Hypertension (DASH) recommends higher intakes of fruits, vegetables, and low-fat dairy foods, alongside limited total fat and sodium intake [9]. It is not strictly categorized as a low-purine diet, nor is it a dietary pattern specifically designed for hyperuricemia. The DASH diet has been verified to reduce sUA levels in hypertensive populations [10]; however, the urate-lowering efficacy of the DASH diet presents significant heterogeneity across different populations, and its therapeutic effects in patients with hyperuricemia or gout remain unclear [11]. Although weight loss diets such as the low-fat diet and Mediterranean diet do not emphasize purine restriction, they have also been observed to lower uric acid levels [12].

Accumulating studies have demonstrated that hyperuricemia is tightly linked to the onset and progression of obesity and other metabolic disorders [5]. Consistently, sUA levels are positively correlated with body mass index (BMI) and visceral fat area (VFA) [13,14]. Uric acid metabolism is intrinsically interconnected with systemic energy metabolism, including lipid and fructose metabolic pathways [15]. Weight loss has been shown to effectively reduce sUA concentrations and decrease the recurrence rate of gout flares [5]. As the final metabolic product of purines—key bioactive molecules involved in the synthesis of ATP, GTP, and coenzyme A—uric acid levels are highly susceptible to dietary purine and energy intake [6]. Based on this metabolic interplay, combined restriction of dietary purine and energy intake via standardized nutritional intervention is hypothesized to be a promising strategy for optimizing gout management.

Current clinical guidelines consistently recommend nutritional therapy as a fundamental component of gout management, and routine dietary health education is widely implemented in clinical practice [16,17]. Nevertheless, it is challenging for most gout patients to achieve clinically meaningful sUA reduction merely through conventional dietary guidance [18]. Existing studies predominantly focus on the epidemiological correlation between single dietary factors and uric acid metabolism. Notably, high-quality empirical studies evaluating the clinical efficacy of standardized, structured, and targeted comprehensive nutritional interventions for gout remain scarce.

In this study, we designed and implemented a structured nutritional intervention with a low-purine, energy-restricted, and balanced diet for gout patients. The present study aimed to evaluate the efficacy of this targeted nutritional therapy in reducing sUA levels and improving concomitant metabolic risk factors. The findings provide credible clinical evidence and novel insights for the comprehensive, standardized non-pharmacological management of gout.

## 2. Materials and Methods

### 2.1. Study Design

**Figure 1:**
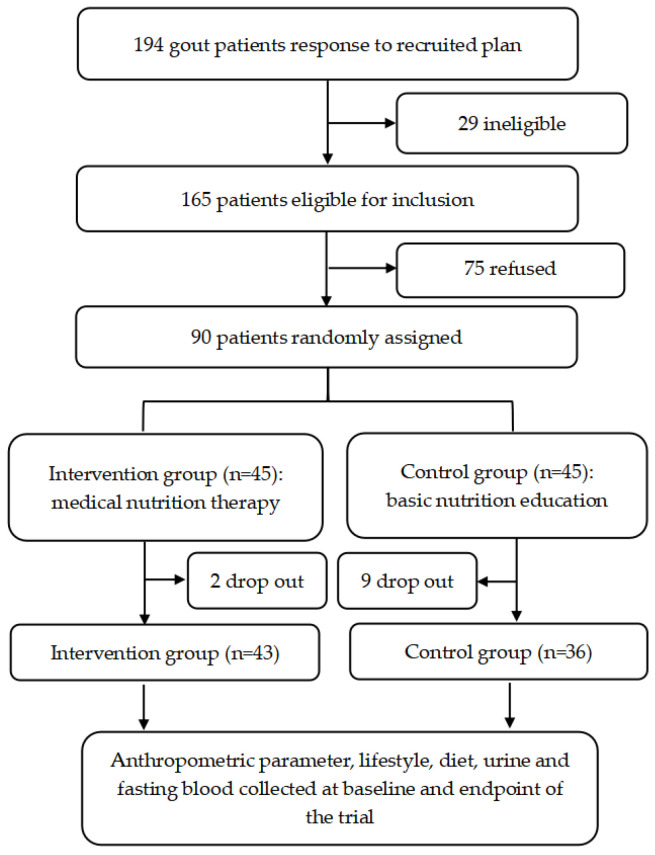
Flow chart of the present trial.

This study was a single-center, parallel-group randomized controlled trial to investigate the therapeutic effect of low-purine and energy-restricted nutritional therapy in male patients with gout. The intervention duration was set at 42 days, which fell within the 4–8-week observation period commonly used in dietary intervention studies [10,11]. The trial was conducted from April 2022 to July 2022 at the Affiliated Hospital of Qingdao University, with changes in serum uric acid (sUA) levels defined as the primary outcome (Figure 1).

This study was approved by the Ethics Committee of the Affiliated Hospital of Qingdao University (Approval No.: QYFYWZLL25519) and was conducted in strict accordance with the ethical principles outlined in the Declaration of Helsinki. The clinical trial was officially registered in the Chinese Clinical Trial Registry (Registration No.: ChiCTR2200059154; available at http://www.chictr.org.cn/) on 26 April 2022. All enrolled participants provided written informed consent prior to study enrollment.

### 2.2. Study Participants

Participants were required to have a confirmed pre-existing clinical diagnosis of gout, which was independently confirmed by an endocrinologist during the study enrollment screening, according to the 2015 American College of Rheumatology/European League Against Rheumatism (ACR/EULAR) Classification Criteria for Gout [19]. This criterion is a comprehensive classification system that incorporates multiple dimensions, including clinical manifestations (e.g., recurrent acute monoarthritis attacks, tophi), laboratory tests (e.g., hyperuricemia, urate crystal identification in synovial fluid), and imaging features (e.g., ultrasound double contour sign, characteristic radiographic erosions), to achieve high specificity and sensitivity for gout diagnosis.

a. Eligibility Criteria

Inclusion Criteria: (1) aged between 18 and 75 years; (2) clinically diagnosed with gout; (3) male gender; (4) no prior history of standardized nutritional intervention for gout before enrollment.

Exclusion Criteria: Participants meeting any of the following conditions were excluded from the study: (1) concurrent use of urate-altering medications other than febuxostat (the basic urate-lowering drug); (2) diagnosis of renal diseases, malignant tumors, or other systemic diseases that interfere with purine and uric acid metabolism; (3) implantation of a cardiac pacemaker (a contraindication for body composition detection); (4) diagnosis of secondary gout.

b. Setting and Recruitment

Participants were consecutively recruited from the specialized gout clinic of the Affiliated Hospital of Qingdao University during the study period from April 2022 to May 2022. There were 16.2 million cases of gout in China, with an age-standardized prevalence rate (ASPR) of 12.3‰ and 3.9‰ in males and females, respectively [20]. Estradiol lowers urate by suppressing the protein levels of urate reabsorptive transporters, urate transporter 1 (URAT1), glucose transporter 9 (GLUT9), and the urate efflux transporter ATP-binding cassette sub-family G member 2 (Abcg2) [21]. Given the low prevalence of gout in women and the substantial influence of estrogen on uric acid metabolism, all participants enrolled in this study were male to avoid potential confounding factors.

### 2.3. Interventions

a. Standard Basic Nutrition Education (for both Control and Intervention groups):

Both the control and intervention groups received unified standard basic nutrition education strictly formulated in accordance with the Chinese Multidisciplinary Expert Consensus on the Diagnosis and Treatment of Hyperuricemia and Related Diseases [22]. All participants were provided with a printed dietary guidance brochure containing standardized dietary principles for gout management. The core educational contents included balanced dietary patterns and rational nutrient restriction: high-purine foods (e.g., animal offal, seafood, and red meat) were strictly limited, while adequate vegetable intake was encouraged. To maintain fluid balance, participants were instructed to drink adequate water to maintain a daily urine output of 2000–3000 mL. Low-fat milk and dairy products were recommended, whereas all sugar-sweetened beverages, including sodas and commercial fruit juices, were prohibited. In terms of lifestyle modification, reduced alcohol and added sugar consumption, as well as minimized dining out, were advocated to stabilize daily nutrient intake. Weekly health education and personalized dietary reminders were consistently implemented to consolidate dietary knowledge and promote behavioral adherence.

b. Medical Nutrition Therapy—Low-Purine, Energy-Restricted, and Balanced Diet (for the Intervention group only):

Based on unified basic nutritional education, participants in the intervention group received a medical nutrition therapy program with a low-purine, energy-restricted, balanced diet. Nutrient requirements were determined according to each participant’s BMI, habitual physical activity level, and basal metabolic rate (BMR) measured via body composition analysis.

The initial energy prescription was set at 25 kcal/kg ideal body weight, with a standardized macronutrient distribution of 50–55% carbohydrates, 25–30% fat, and 15–20% protein. The daily protein intake was standardized to 1.2 g/kg of ideal body weight [23], with further fine-tuning performed according to individual BMR and physical activity intensity. For participants with moderate-to-vigorous physical activity, total daily energy intake was appropriately increased by 20%. In the intervention group (*n* = 45), stratified energy prescription regimens were scientifically formulated based on the individually calculated energy requirements as described above. Participants were assigned to corresponding energy tiers in accordance with their computed energy demands. These regimens comprised: 2000 kcal/d (*n* = 6; target range: 1900–2099 kcal/d), 1800 kcal/d (*n* = 24; target range: 1700–1899 kcal/d), 1600 kcal/d (*n* = 12; target range: 1500–1699 kcal/d), and 1400 kcal/d (*n* = 3; target range: 1300–1499 kcal/d). This individualized energy restriction achieved a moderate energy deficit, ranging from 10% to 35% relative to participants’ habitual intake. To preserve lean mass during energy restriction, the relative proportion of dietary protein was maintained at an adequate level. Concurrently, daily total dietary purine intake was limited to a maximum of 200 mg (during acute gout attacks) or 600 mg (during remission). All nutritional targets were translated into meal schemes and a standardized 7-day rotating dietary recipe (Supplementary Table S1A–H). Before the formal intervention, each participant completed a 30 min one-on-one dietary counseling session with a professional dietitian to clarify standardized food selection, accurate portion control, and healthy cooking methods, ensuring full understanding and high adherence to the prescribed dietary protocol throughout the intervention period.

c. Strategies to Ensure Similar Care and Adherence:

All participants were required to maintain their usual physical activity levels for the entire 42-day study period. Throughout the intervention, participants were strictly prohibited from altering the type or dosage of their uric acid-lowering medications. Any exceptional circumstance requiring medication adjustment would lead to the participant’s withdrawal from the study.

At baseline, each participant received a one-on-one face-to-face dietary consultation from a specialized nutrition physician. Standardized weekly remote follow-ups were subsequently conducted via the WeChat app (v8.0.58) throughout the intervention period [24]. During each follow-up session, nutritionists delivered continuous health education, monitored dietary adherence, and provided timely individualized correction of inappropriate dietary behaviors.

### 2.4. Outcomes

Primary Outcome: The primary outcome was the change in sUA level from baseline to day 42.

Secondary Outcomes: Secondary outcomes included changes in body composition, dietary intake, and other metabolic profiles assessed at baseline and day 42.

### 2.5. Sample Size

Sample size calculation was performed based on the primary outcome (change in sUA level). According to previous clinical evidence, a minimal detectable between-group difference of 0.48 mg/dL in sUA variation was assumed [25]. A total of 30 participants per group were required to achieve a statistical power of 80% with a two-tailed significance level of 0.05. Considering an expected 20% participant dropout rate during the 42-day intervention, a minimum of 38 subjects per group was required to ensure a valid statistical analysis of the primary outcome.

### 2.6. Randomization

A computer-generated block randomization (blocks of varying sizes) was used to allocate 90 participants in a 1:1 ratio to the intervention group (*n* = 45) and the control group (*n* = 45).

The randomization sequence was generated by an independent statistician who was not involved in recruitment or intervention. Allocation was concealed using sequentially numbered, opaque, sealed envelopes. Envelopes were opened only after the participants had completed all baseline assessments and provided informed consent.

### 2.7. Blinding

Due to the nature of the dietary intervention, this study employed an open-label design, where blinding was not implemented. Both participants and the dietitians providing the dietary intervention were aware of the group assignments. However, outcome assessors (laboratory personnel performing biochemical analyses) were blinded to group assignment. The statistician who performed the primary analyses remained blinded until those analyses were completed.

### 2.8. Data Collection and Measurement Methods

At baseline, all participants underwent demographic data collection, dietary intake assessment, body composition analysis, and collection of fasting blood and urine samples for biochemical analyses. At the study endpoint (Day 42), all baseline measurements were precisely repeated.

Dietary Adherence Monitoring: Dietary intake was systematically evaluated using a validated semi-quantitative food frequency questionnaire (SQFFQ) [26], combined with detailed 3-day dietary records (two weekdays and one weekend day) [27]. Participants received verbal and written instructions on how to accurately record all food and beverages consumed, including preparation methods and brand names. They were required to submit photographs of their daily meals during the designated 3-day recording (two weekdays and one weekend day) weekly via the WeChat app online. Macronutrient and purine intakes were quantitatively analyzed using the Traditional Chinese and Western Medicine Combined Nutrition System (NCCW, version 11.0) based on the photographs [28].

All measurements were performed by trained personnel following standard protocols. Height and weight were taken to the nearest 0.1 cm and 0.1 kg using a digital stadiometer (InBody BSM370, InBody Co., Ltd., Seoul, Republic of Korea), respectively, with participants barefoot and in light clothing. Waist and hip circumferences were measured to the nearest 0.1 cm using a non-stretchable tape at standard anatomical sites. Blood pressure was measured after a 5 min seated rest using an automated sphygmomanometer (Omron HEM-7130, Omron Healthcare Co., Ltd., Kyoto, Japan) with an appropriately sized cuff [29]. Two consecutive readings were recorded, and their average was used for systolic and diastolic blood pressure (mmHg) [30].

Body composition was measured via bioelectrical impedance analysis (BIA) using a validated commercial analyzer (InBody S10, InBody Co., Ltd., Seoul, Republic of Korea) following the manufacturer’s standard protocol. Outcome variables included Fat Mass (FM), Skeletal Muscle Mass (SMM), Percentage Body Fat (PBF%), and VFA. Before measurement, participants were instructed to fast overnight and empty their bladders; they were required to remove all metal accessories and wear only light clothing during the test [31].

For biochemical analyses, fasting venous blood and first-morning urine samples were collected after a minimum of an 8 h overnight fast. All samples were processed within 30 min of collection: blood samples were centrifuged at 3000× *g* for 10 min at 4 °C using a refrigerated centrifuge (Thermo Scientific Sorvall ST 16R, Thermo Fisher Scientific, Osterode, Germany) to separate serum, and urine samples were centrifuged to remove sediment. All processed samples were aliquoted and stored at −80 °C in an ultra-low temperature freezer (Haier DW-86L626, Haier Biomedical, Qingdao, China) until testing, which was conducted by trained technicians using standardized commercial kits on an automated clinical biochemistry analyzer (Hitachi 7100, Hitachi High-Tech Corporation, Tokyo, Japan) [32].

### 2.9. Statistical Analysis

All intervention effect analyses were performed on the intention-to-treat analysis of the primary outcomes. This set included participants who completed the intervention, achieved ≥80% adherence, had no major protocol violations, and provided complete 42-day outcome data. Continuous variables were presented as mean ± SD or median (IQR), and categorical variables as counts (percentages). Baseline characteristics were compared between groups using independent *t*-tests for normally distributed continuous data, Mann–Whitney U tests for non-normally distributed continuous data, and chi-square tests for categorical data. Paired *t*-tests (or Wilcoxon signed-rank tests for non-normally distributed changes) were used to evaluate within-group changes. Inter-group differences in outcomes were compared using general linear models (GLM). Multivariable-adjusted GLM analyses also controlled for potential confounders: age, tobacco and alcohol consumption status, daily dietary calorie, macronutrient, and purine intake, baseline BMI, baseline blood pressure, sUA, concomitant metabolic diseases, and ongoing medication therapy. All statistical tests were two-tailed, with significance set at *p* < 0.05. Analyses were performed using Stata 15.0 software (Stata Corp, College Station, TX, USA).

## 3. Results

### 3.1. Baseline Characteristics of Study Participants

A total of 90 eligible male patients with gout were enrolled in this study between April and July 2022 and were randomly assigned to either the intervention group (*n* = 45) or the control group (*n* = 45). The primary outcome analysis was performed on an ITT basis, encompassing all 90 randomized participants. During the 42-day intervention period, two participants in the intervention group and nine participants in the control group withdrew voluntarily for personal reasons, primarily due to failure to attend scheduled follow-up assessments. To address these withdrawals and subsequent missing data, missing values for primary outcomes were imputed using the Last Observation Carried Forward (LOCF) method. While these withdrawals meant that 43 and 36 participants, respectively, completed all scheduled follow-up assessments and provided observed data at the final time point, the analytical approach ensured that data from all 90 randomized subjects were included in the final analysis (Figure 1).

Baseline demographic, clinical, and biochemical characteristics were well balanced between the two groups, with no significant intergroup differences observed except for blood urea nitrogen (BUN) (all other *p* > 0.05) (Table 1). No statistically significant differences were found in age (*p* = 0.228) and BMI (*p* = 0.606) at baseline. Similarly, key clinical indicators, including sUA levels (*p* = 0.843) and renal function (estimated glomerular filtration rate: 96.0 vs. 96.8 mL/min/1.73 m^2^, *p* = 0.932), as well as lipid profile parameters, were comparable between groups. In terms of body composition, VFA (*p* = 0.203) and SMM (*p* = 0.141) showed no significant baseline differences. The prevalence of comorbidities, including hypertension, hyperlipidemia, and non-alcoholic fatty liver disease, was similar across the two groups. Additionally, the baseline daily dosage of febuxostat was comparable between the intervention and control groups (31.6 mg vs. 30.7 mg, *p* = 0.842). These findings confirmed a homogeneous and balanced baseline clinical status for all enrolled participants.

Baseline habitual dietary intake was also comparable between the two groups (Table 2). Vegetable intake was insufficient in both groups (intervention group: 282.5 ± 209.8 g/d; control group: 313.5 ± 248.6 g/d), which was below the daily recommended intake of 300–500 g/d according to the Chinese Dietary Guidelines [33].

Baseline daily nutrient intake was fully matched between the two groups in terms of total energy, carbohydrates, fat, protein, and total purine content (Table 3). Both groups exhibited excessive daily energy intake, with average values of 2579.3 ± 702.4 kcal/d in the intervention group and 2755.1 ± 792.5 kcal/d in the control group. These levels substantially exceeded the recommended intake specified in the Chinese Residents’ Dietary Nutrient Reference Intakes, indicating a prevalent state of positive energy balance among the enrolled gout patients. Moreover, the baseline daily purine intake was excessively high in both groups (intervention group: 644.2 ± 401.0 mg/d; control group: 798.8 ± 605.0 mg/d), both exceeding the threshold of 600 mg/d and confirming an overall high-purine dietary pattern at baseline.

### 3.2. Primary Outcome: Changes in sUA Levels

**Figure 2:**
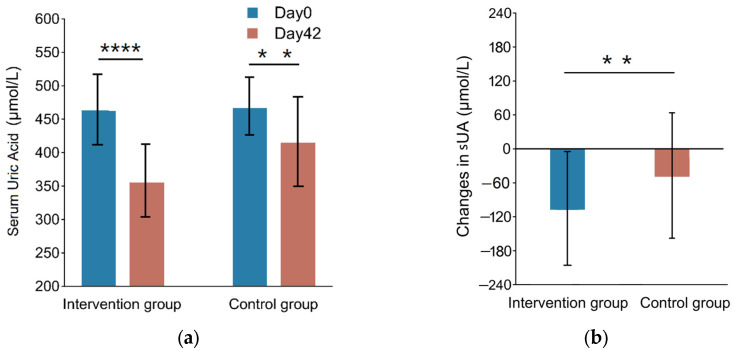
Effects of LPEB diet on sUA. (**a**) Comparison of sUA levels on Day 0 and after 42 days of intervention in the control and intervention groups; (**b**) Changes in sUA from Day 0 to Day 42 in the intervention and control groups. Intervention group: participants receiving the LPEB diet; Control group: participants receiving the basic nutritional education. Data are mean ± SD; ** *p* < 0.01, **** *p* < 0.001.

After the 42-day intervention, the intervention group exhibited a significant reduction in sUA levels, with a mean decrease of 112.4 µmol/L (*p* < 0.001) relative to baseline (Figure 2a). In comparison, the control group achieved a reduction of 47.0 µmol/L (*p* = 0.001). Multivariable-adjusted analysis confirmed that the intervention group had a significantly greater sUA-lowering effect than the control group (mean difference: −65.4, 95% CI: −116.1 to −18.2, *p* = 0.007) (Figure 2b). PP analysis further supported the above effects (Supplementary Table S2). The sUA target achievement rate in the intervention group increased from 11.1% at baseline to 71.1% at the endpoint, whereas in the control group it rose from 11.1% to 40%. Collectively, these results demonstrated that the structured low-purine, energy-restricted, balanced diet exerted superior efficacy in reducing sUA levels compared with basic nutrition education alone.

### 3.3. Effects of LPEB Diet on Food Group Intake

This intervention followed core gout dietary principles: higher plant food intake, optimized high-quality protein sources to lower total dietary purine load, and limiting refined sugars, dietary fat, and daily total energy intake. Table 4 summarizes major food group intake changes in both groups after the 42-day intervention.

The medical nutrition therapy effectively reshaped the dietary patterns of participants in the intervention group. The within-group analysis showed significant reductions in the consumption of desserts (*p* = 0.016), red meats (*p* = 0.001), poultry (*p* = 0.030), cereals (*p* < 0.001), fruits (*p* = 0.047), and cooking oil (*p* < 0.001). Meanwhile, dietary intakes of beneficial foods, including vegetables, eggs, and dairy products, were significantly increased (all *p* < 0.001). However, in terms of energy contribution, no significant changes were observed for desserts, poultry, cereals, fruits, and cooking oil. Conversely, a significant decrease was found for red meat (*p* = 0.006), while the energy contribution from vegetables, eggs, and dairy products increased significantly (all *p* < 0.001).

Intergroup comparison further verified significant differences in dietary modification effects between the two groups. Compared with the control group, the intervention group showed a greater reduction in red meat, fruit, and oil intake (both *p* < 0.05). In terms of energy contribution, fruit and oil intake did not differ significantly between the two groups. The intervention group demonstrated more prominent increases in vegetables (*p* < 0.001), eggs (*p* < 0.001), and dairy products (*p* = 0.047) consumption. Concurrently, the energy contribution from these three food categories also significantly increased in the intervention group (all *p* < 0.05).

In summary, the low-purine, energy-restricted, and balanced diet successfully optimized the dietary structure of gout patients. The intervention effectively reduced the intake of high-purine and high-fat foods (represented by red meat and oil) while substantially increasing the consumption of vegetables, eggs, and dairy products, thereby establishing a healthier and more gout-friendly dietary pattern.

### 3.4. Effects of LPEB Diet on Nutrient Intake

Changes in daily energy intake and major nutrient profiles (including carbohydrates, fat, protein, and total purine) after the intervention are summarized in Table 5. Although some participants did not strictly adhere to the prescribed protocol, the medical nutrition therapy induced substantial improvements in overall nutrient intake in the intervention group. Within-group analysis demonstrated a significant reduction in daily energy intake in the intervention group, decreasing from 2579.3 ± 702.4 kcal/d at baseline to 2024.2 ± 378.0 kcal/d at the endpoint (*p* < 0.001), with a mean reduction of 555.0 kcal/d. Similarly, the intake of carbohydrates, fat, and protein was significantly decreased after the 42-day intervention (all *p* < 0.001). Notably, purine intake in the intervention group was markedly reduced from 644.2 ± 401.0 mg/d to 399.2 ± 197.1 mg/d (*p* < 0.001), corresponding to a mean purine intake reduction of 245.0 mg/d. In contrast, the control group exhibited minimal alterations in nutrient profiles during the study period, with significant reductions observed only in dietary energy (*p* = 0.007) and fat intake (*p* = 0.030); no significant changes in carbohydrate, protein, or purine intake were detected (all *p* > 0.05).

Further intergroup comparison confirmed that the intervention group achieved significantly greater reductions in total energy, carbohydrates, fat, protein, and purine intake than the control group. However, no statistically significant change was observed in the macronutrient energy contribution of the intervention group compared to the control group.

These findings indicated that the low-purine, energy-restricted, balanced diet medical nutrition therapy effectively regulated macronutrient and purine intake, successfully correcting the high-energy and high-purine dietary patterns prevalent among gout patients at baseline.

### 3.5. Effect of LPEB Diet on Anthropometric Parameters

The optimized macronutrient profiles and controlled energy intake achieved via nutritional intervention contributed to significant improvements in anthropometric and hemodynamic parameters (Table 6). Following the 42-day intervention, the intervention group presented a significant within-group reduction in body weight, with a mean decrease of 1.63 kg (from 83.3 ± 12.1 kg to 81.6 ± 11.9 kg, *p* < 0.001). Accordingly, BMI was significantly reduced by 0.50 kg/m^2^ (from 27.4 ± 3.2 kg/m^2^ to 26.9 ± 3.1 kg/m^2^, *p* < 0.001). In comparison, the control group showed no remarkable changes in body weight or BMI throughout the study period. Intergroup comparison verified that the intervention group yielded significantly greater reductions in body weight (−1.63 kg vs. 0.14 kg, mean difference: −1.77, 95% CI: −2.35 to −0.78, *p* < 0.001) and BMI (−0.50 kg/m^2^ vs. 0.05 kg/m^2^, mean difference: −0.55, 95% CI: −0.75 to −0.25, *p* < 0.001) relative to the control group.

In terms of blood pressure indicators, the intervention group achieved significant within-group improvements in systolic blood pressure (SBP) and diastolic blood pressure (DBP). SBP decreased significantly from 134.6 ± 19.9 mmHg to 131.0 ± 17.7 mmHg (*p* = 0.044), while DBP declined from 85.9 ± 12.1 mmHg to 82.6 ± 12.0 mmHg (*p* = 0.007). Conversely, no significant longitudinal changes in SBP or DBP were observed in the control group. Despite the notable within-group improvements in blood pressure in the intervention group, the between-group differences in the magnitude of blood pressure reduction did not reach statistical significance.

### 3.6. Effect of LPEB Diet on Body Composition Parameters

The 42-day intervention showed significant beneficial alterations in body composition profiles in the intervention group (Table 7). Within-group analyses demonstrated substantial reductions in multiple adiposity-related indicators. Total body fat mass (FAT) decreased significantly from 25.7 ± 5.6 kg to 23.3 ± 5.1 kg (*p* < 0.001), while percent body fat (PBF) declined from 30.7 ± 4.3% to 28.7 ± 4.6% (*p* < 0.001). Similarly, waist circumference (WC) was reduced from 97.6 ± 9.7 cm to 95.0 ± 8.9 cm (*p* < 0.001), and VFA was markedly decreased from 116.1 ± 28.5 cm^2^ to 104.0 ± 26.0 cm^2^ (*p* < 0.001). In contrast, none of these body composition parameters exhibited significant longitudinal changes in the control group throughout the intervention period.

Intergroup comparison further confirmed that the intervention group achieved significantly greater improvements in fat-related anthropometric indices than the control group. The mean reduction in FAT was 2.39 kg in the intervention group, whereas a slight increase of 0.58 kg was observed in the control group (*p* < 0.001). Significant between-group differences were also identified for changes in PBF (−1.92% vs. 0.58%; *p* < 0.001), WC (−2.61 cm vs. 1.03 cm; *p* < 0.001), and VFA (−12.1 cm^2^ vs. 3.56 cm^2^; *p* < 0.001). No significant intergroup differences were detected in the changes in non-adipose parameters, including protein, mineral, SMM, body cell mass, bone mineral content, and basal metabolic rate.

Collectively, these results indicated that the low-purine, energy-restricted, balanced medical nutrition therapy effectively alleviated systemic fat accumulation, particularly reducing visceral fat deposition and ameliorating central obesity in gout patients, while successfully preserving lean body mass and fundamental basal metabolic characteristics.

### 3.7. Effects of LPEB Diet on Biochemical Parameters

Changes in blood and urinary biochemical profiles following the 42-day intervention are summarized in Table 8. Collectively, the intervention group achieved substantial improvements in multiple hepatic, glycolipid, and renal biochemical markers relative to the control group.

For hepatic function indicators, alanine aminotransferase (ALT) levels decreased significantly by a mean of 4.49 U/L in the intervention group (*p* = 0.009). There was no significant difference in ALT between the two groups. In contrast, no significant within-group change was observed in aspartate aminotransferase (AST) levels in the intervention group, and no significant intergroup difference in AST alteration was detected between the two groups.

In terms of glycolipid metabolic profiles, the intervention group exhibited a significant reduction in serum triglyceride (TG) levels, decreasing from 2.23 ± 1.05 mmol/L to 1.83 ± 0.93 mmol/L (*p* < 0.001), with a mean change of −0.40 mmol/L. The control group showed no detectable change in TG levels during the intervention, and the magnitude of TG reduction differed significantly between the two groups (*p* = 0.023). Fasting blood glucose (BG) levels and total cholesterol (TC) levels remained stable in both groups throughout the study period, with no remarkable within-group or intergroup changes.

Regarding renal function and uric acid metabolism, the intervention group demonstrated significant improvements in multiple renal biomarkers. Estimated glomerular filtration rate (GFR) was significantly elevated after nutritional intervention (*p* = 0.019), while no obvious change was found in the control group, yielding a significant intergroup difference in GFR variation (*p* = 0.038). Serum creatinine (CREA) levels were markedly reduced in the intervention group (*p* = 0.015) but remained unchanged in the control group, with a significant between-group difference observed (*p* = 0.016). Conversely, urinary creatinine (CREA-U) levels increased significantly in the intervention group (*p* = 0.018) but not in the control group, with a marginally significant intergroup difference (*p* = 0.060). Additionally, blood urea nitrogen (BUN) levels increased significantly in the intervention group (*p* = 0.020), while BUN levels did not change significantly in the control group (*p* = 0.070), resulting in a highly significant intergroup difference in BUN changes (*p* = 0.002).

The concurrent improvements in renal indicators, including decreased serum CREA, elevated CREA-U and GFR, and moderately increased BUN, indicated enhanced protein metabolic turnover and improved renal excretory function in the intervention group, without additional renal burden. Furthermore, the fractional excretion of uric acid (FEUA) in the intervention group increased from 3.98 ± 2.16% to 4.85 ± 3.87%, with a mean change of 0.87 ± 2.60, while there was no change in the control group. The improvement in FEUA was significantly greater in the intervention group than in the control group (*p* = 0.001). The findings of the present study indicated that a low-purine, energy-restricted, balanced dietary intervention enhanced uric acid excretion and thereby reduced sUA levels. No significant longitudinal or intergroup differences were observed in urinary uric acid concentrations and urinary pH in either group.

### 3.8. Adverse Effects or Harms

Participant safety was systematically monitored throughout the study via weekly online interviews. No adverse events (including, but not limited to, nausea, headache, allergic reactions) were observed during the study.

## 4. Discussion

The present randomized controlled trial demonstrated that a structured low-purine, energy-restricted, balanced dietary intervention significantly improved urate metabolism and overall metabolic profiles in male patients with gout. The intervention group exhibited a substantial reduction in sUA and a remarkable elevation in the FEUA, indicating optimized systemic urate homeostasis following nutritional modification. Concurrently, multiple hepatic, cardiometabolic, and anthropometric risk indicators, including BMI, blood pressure, TG, total cholesterol, serum creatinine, body fat percentage, and VFA, were markedly improved, accompanied by an increase in glomerular filtration rate. Collectively, these findings indicated that standardized medical nutrition therapy targeting exogenous purine load and energy balance could simultaneously ameliorate hyperuricemia and its concomitant cardiometabolic complications.

This intervention demonstrated superior efficacy beyond conventional passive nutritional education, primarily through its innovative ‘substitution and optimization’ strategy. This approach effectively lowered the exogenous purine load by strategically replacing purine-dense red meats with high-biological-value proteins (dairy and eggs). Importantly, this purine reduction was accomplished without the typical drawbacks of energy-restricted diets, such as negative nitrogen balance, which often exacerbates muscle catabolism and can contribute to secondary hyperuricemia.

A significant reduction in sUA was observed, with a decrease of 112.4 μmol/L, from an initial value of 470.6 μmol/L to 358.2 μmol/L. This reduction is of considerable clinical significance, as it is typically sufficient to enable gout patients to achieve the treatment target sUA levels recommended by international guidelines (<360 μmol/L) [34]. Achieving and maintaining sUA levels below 360 µmol/L (6 mg/dL) is critical as it represents the saturation point of urate in plasma, effectively inhibiting the formation of new monosodium urate crystals [35]. Consequently, this therapeutic target is essential for preventing acute gout flares, promoting the dissolution of existing tophi to reverse joint damage, and reducing the risk of uric acid nephrolithiasis. Moreover, it may offer broader protective effects for renal and cardiovascular health, considering the common comorbidities associated with hyperuricemia. Ultimately, this stringent control is crucial for improving patient outcomes and preventing long-term complications of gout.

Furthermore, by maintaining adequate carbohydrate intake, our balanced dietary regimen circumvented the urate retention commonly seen with ketogenic or severely carbohydrate-restricted diets, which is mediated by competitive inhibition from ketone bodies. This not only prevented ketosis-induced complications but also facilitated a sustainable rate of weight loss, a crucial element for ensuring long-term patient adherence and metabolic homeostasis.

Beyond reducing the exogenous purine load, the intervention significantly enhanced renal uric acid clearance, as evidenced by elevated FEUA and GFR. This improvement is closely associated with the substantial increase in vegetable intake observed in the intervention group. A cohort study demonstrated a significant negative association between a high-vegetable plant-based dietary pattern and sUA levels [36]. The beneficial effects of vegetables are likely mediated not only by their low purine content but also by their diverse bioactive phytochemical constituents, such as flavonoids and polyphenols [37]. Plant-derived active compounds, such as saponins, coumarins, and lignin, have effects on lowering sUA [38]. Preclinical studies have verified that vegetable-derived active ingredients (e.g., celery seed extract) suppress hepatic activities of xanthine dehydrogenase and xanthine oxidase, thereby reducing serum urate accumulation [39,40]. Moreover, these phytochemicals facilitate renal urate excretion by modulating key renal urate transporters (URAT1 and GLUT9) and attenuating tubular urate reabsorption. For example, apigenin-7-O-β-D-glucuronide concurrently inhibits hepatic uric acid production, regulates renal urate transport, and alleviates hyperuricemia-related renal injury [41]. This “renal-protective” dietary effect explains why sUA levels decreased, while GFR and FEUA improved, suggesting a reversal of the subclinical renal impairment often seen in chronic gout patients.

Beyond purine regulation, the synergistic benefits of energy restriction and adiposity improvement represent another core mechanistic highlight of this intervention. Obesity is an independent risk factor for gout onset and progression [42]. Population-based data from the National Health and Nutrition Examination Survey (NHANES) have confirmed that excessive weight gain and obesity increase gout risk, whereas intentional weight loss to a normal body weight significantly reduces gout susceptibility (OR = 0.41, *p* = 0.008) [43]. Clinical interventional studies further support this causal relationship, demonstrating that energy restriction effectively decreases circulating sUA levels in obese individuals [12]. A bidirectional pathological interaction exists between hyperuricemia and adiposity: elevated sUA promotes lipogenesis and pro-inflammatory phenotypic transformation in adipocytes to facilitate fat accumulation [44], whereas obesity exacerbates urate overproduction and renal urate retention. This reciprocal vicious cycle provides a fundamental theoretical basis for the integrated low-purine and energy-restricted intervention applied in the current study. At baseline, enrolled participants exhibited a high burden of central overweight or central obesity, with VFA substantially exceeding normal clinical thresholds. Following the 42-day intervention, the intervention group achieved selective adipose tissue reduction, highlighted by a 12.1 cm^2^ decrease in VFA and a 2.4 kg reduction in total fat mass, while preserving lean body mass. Therefore, intervention-induced visceral fat loss alleviated adipose inflammation and improved insulin sensitivity, disrupting the vicious cycle of urate accumulation, adipocyte inflammation, and insulin resistance, making it a crucial auxiliary target for non-pharmacological gout management.

Beyond optimized urate homeostasis, the intervention produced comprehensive systemic metabolic improvements. Significant reductions in systolic (*p* = 0.044) and diastolic blood pressure (*p* = 0.007) further demonstrate the cardiovascular protective effects of this dietary pattern, which may be mediated by reduced urate burden, attenuated systemic inflammation, improved endothelial function, and suppressed renin–angiotensin–aldosterone system overactivation [45].

Several limitations of the present study should be acknowledged. First, this study only had a 42-day observation period, which precluded us from assessing the long-term efficacy and safety of the tested intervention. Due to the short intervention period, the impact of this dietary pattern on the frequency of gout attacks was not assessed. Second, all participants enrolled in this study were male; we made this restriction given the low prevalence of gout in females and the well-documented effect of estrogen on uric acid metabolism, with a gender-stratified randomization design to avoid confounding. Although randomization eliminates gender-related confounding in our effect estimates, the lack of female participants significantly limited the generalizability of our findings to the entire gout population, which was a major limitation of this study. Third, the usage of emergency medications was not statistically recorded. Fourth, owing to the nature of the dietary intervention, blinding was not feasible in this study, which constituted a limitation.

**Figure 3:**
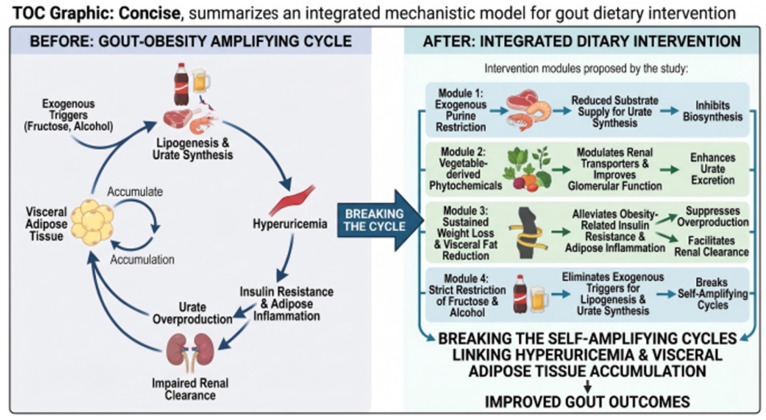
The integrated mechanistic model for gout dietary intervention.

Collectively, this study proposes an integrated mechanistic model for gout dietary intervention (Figure 3). Exogenous purine restriction effectively reduces the substrate supply for uric acid synthesis, thereby inhibiting its biosynthesis, while vegetable-derived phytochemicals modulate renal urate transporters and improve glomerular function to enhance urate excretion. Sustained weight loss and visceral fat reduction alleviate obesity-related insulin resistance and adipose inflammation, further suppressing urate overproduction and facilitating renal clearance. Strict restriction of fructose and alcohol eliminates exogenous triggers for lipogenesis and urate synthesis, ultimately breaking the self-amplifying cycles linking hyperuricemia and visceral adipose tissue accumulation. These metabolic improvements represent systematic multi-target regulatory effects rather than isolated single-index changes. The 42-day intervention period is relatively short; thus, multi-omics studies (metabolomics and proteomics) are warranted to evaluate long-term dietary adherence and identify vegetable-derived phytochemical targets that regulate urate anabolism and excretion. Ultimately, this integrated nutritional intervention provided an optimized, highly translational non-pharmacological strategy for the routine clinical management of gout and its related metabolic comorbidities.

## 5. Conclusions

The present 42-day standardized intervention demonstrates that gout patients achieve notable improvements across multiple critical indicators, including serum uric acid levels, body weight, visceral fat accumulation, lipid profiles, and hepatic and renal metabolic parameters. These findings verify metabolic advantages of integrated low-purine and energy-restricted dietary management. Consequently, this non-pharmacological nutritional strategy provides an optimized intervention scheme for routine clinical gout management.

## Figures and Tables

**Table 1:** Characteristics in baseline.

Basic Characteristics	Intervention Group (*n* = 45)	Control Group (*n* = 45)	*p*-Value
Demographic parameter			
Age, y	44.0 ± 12.9	47.1 ± 11.3	0.228
BMI, kg/m^2^	27.4 ± 3.2	26.8 ± 3.3	0.418
Weight, kg	83.9 ± 12.6	80.6 ± 11.7	0.313
Height, cm	174.1 ± 6.1	173.2 ± 6.0	0.097
SBP, mmHg	134.6 ± 19.9	133.6 ± 15.2	0.794
DBP, mmHg	85.9 ± 12.1	86.1 ± 10.0	0.939
Current smoker (%)	17(37.8%)	20(44.4%)	0.438
Current drinker (%)	16(35.6%)	15(33.3%)	0.894
Clinical parameters			
ALT, U/L	35.9 ± 20.8	40.6 ± 24.4	0.329
AST, U/L	26.0 ± 12.1	26.3 ± 9.8	0.824
TG, mmol/L	2.23 ± 1.05	1.92 ± 1.03	0.164
TC, mmol/L	5.12 ± 1.09	4.93 ± 1.02	0.402
BG, mmol/L	5.79 ± 0.92	5.55 ± 0.98	0.241
UA, µmol/L	470.5 ± 102.3	466.6 ± 87.1	0.843
BUN, mmol/L	4.49 ± 1.52	5.08 ± 1.21	0.007
CREA, µmol/L	85.2 ± 17.2	81.9 ± 16.7	0.359
GFR, mL/min/1.73 m^2^	96.0 ± 22.5	96.8 ± 22.5	0.932
CREA-U, mmol/L	10.4 ± 8.3	11.6 ± 6.1	0.445
UA-U, mmol/L	1.99 ± 1.29	2.22 ± 1.23	0.395
FEUA, %	3.98 ± 2.16	4.24 ± 1.82	0.542
pH-U	5.82 ± 0.64	5.93 ± 0.70	0.468
Body composition parameters			
TBW, kg	42.5 ± 6.5	41.7 ± 5.4	0.500
Protein, kg	11.4 ± 1.8	11.1 ± 1.5	0.495
Mineral, kg	3.90 ± 0.66	3.77 ± 0.56	0.302
FAT, kg	25.7 ± 5.6	23.7 ± 7.6	0.163
FFM, kg	57.8 ± 8.9	56.6 ± 7.4	0.479
SMM, kg	32.4 ± 5.4	31.6 ± 4.5	0.141
PBF, %	30.7 ± 4.3	29.0 ± 5.8	0.124
BCM, kg	37.8 ± 5.9	36.9 ± 4.9	0.478
BMC, kg	3.20 ± 0.56	3.09 ± 0.45	0.293
WC, cm	97.6 ± 9.7	94.9 ± 11.8	0.228
VFA, cm^2^	116.1 ± 28.5	106.8 ± 39.5	0.203
BMR, kcal	1619.0 ± 192.5	1592.2± 160.4	0.474
Other metabolic-related diseases			
Type 2 diabetes, n (%)	3 (6.7%)	2 (4.4%)	0.627
Hypertension, n (%)	14 (31.1%)	14 (31.1%)	1.000
Hyperlipidemia, n (%)	18 (40%)	17 (37.8%)	0.873
NAFLD, n (%)	7 (15.6%)	6 (13.3%)	0.763
Current medical treatment of gout			
Febuxostat, mg	31.6 ± 18.8	30.7 ± 23.2	0.842

Abbreviation: SBP, systolic blood pressure; DBP, diastolic blood pressure; ALT, alanine aminotransferase; AST, aspartate aminotransferase; TC, total cholesterol; TG, triglyceride; BG, blood glucose; UA, uric acid; BUN, blood urea nitrogen; CREA, creatinine; GFR, glomerular filtration rate; CREA-U, urinary creatinine; UA-U, urinary uric acid; FEUA, fraction excretion of uric acid, calculated as (urinary uric acid × serum creatinine)/(serum uric acid × urinary creatinine) × 100%; pH-U, urine pH. TBW, total body water; FFM, fat-free mass; SMM, skeletal muscle mass; PBF, percent body fat; BCM, body cell mass; BMC, bone mineral content; WC, waist circumference; VFA, visceral fat area; BMR, basal metabolic rate. NAFLD, non-alcoholic fatty liver disease.

**Table 2:** Food intake at baseline.

Foods	Intervention Group (*n* = 45)	Control Group (*n* = 45)	*p*-Value
Non-shellfish seafood, g	2.0 (0.0, 15.0)	2.5 (0.0, 32.5)	0.324
Dessert, g	10.0 (0.0, 30.0)	10.0 (0.0, 36.7)	0.856
Red meat, g	50.0 (28.8, 100.0)	50.0 (25.0, 150.0)	0.855
Poultry, g	13.8 (0.0, 41.3)	15.0 (0.0, 62.5)	0.401
Cereals, g	341.3 ± 125.5	363.2 ± 127.0	0.444
Soy products, g	10.0 (0.0, 30.0)	7.5 (0.0, 36.3)	0.891
Mushrooms, g	3.0 (0.0, 3.0)	0.0 (0.0, 0.0)	0.729
Fruit, g	250.0 (19.5, 250.0)	240.0 (0.0, 240.0)	0.339
Vegetables, g	282.5 ± 209.8	313.5 ± 248.6	0.541
Eggs, g	57.0 (0.0, 60.0)	60.0 (0.0, 81.0)	0.988
Dairy products, g	75.0 (0.0, 250.0)	25.0 (0.0, 210.0)	0.334
Oil, g	49.2 ± 13.2	49.1 ± 13.0	0.970

Food items, including shellfish, offal, broth, fish roe, sugar-sweetened beverages, and alcoholic beverages, were not presented in the baseline dietary intake table as over 75% of participants reported no consumption; however, their consumption data were included in the calculation of total nutrient intake.

**Table 3:** Daily nutrient intake at baseline.

Nutrients	Intervention Group (*n* = 45)	Control Group (*n* = 45)	*p*-Value
Energy, kcal/d	2579.3 ± 702.4	2755.1 ± 792.5	0.267
Carbohydrate, g/d	361.2 ± 121.3	360.3 ± 110.8	0.970
Fat, g/d	77.2 ± 17.5	80.9 ± 24.9	0.415
Protein, g/d	94.3 ± 37.1	108.0 ± 53.2	0.159
Purine, mg/d	644.2 ± 401.0	798.8 ± 605.0	0.156

**Table 4:** Effects of LPEB diet on food intake.

Time	Intervention Group (*n* = 45)				Control Group (*n* = 45)				Intervention Effect Estimate (95%CI)		*p*-Value	
	Foods, g	*p*-Time ^1^	% of Energy	*p*-Time ^2^	Foods, g	*p*-Time ^1^	% of Energy	*p*-Time ^2^	Foods, g	% of Energy	Foods	% of Energy
Non-shellfish seafood												
Day0	2.5 (0.0, 15.0)	0.224	0.1 (0.0, 0.6)	0.688	2.5 (0.0, 35.0)	0.238	0.1 (0.0, 1.0)	0.944			0.666	0.657
Day42	1.5 (0.0, 12.5)		0.1 (0.0, 0.5)		2.5 (0.0, 25)		0.1 (0.0, 0.8)				0.175	0.700
Change	0.0 (−5.0, 2.5)		0.0 (−0.3, −0.2)		0.0 (−2.5, 0.0)		0.0 (−0.1, 0.0)		−2.4 (−19.3 to 9.2)	−0.7 (−0.8 to 0.4)	0.487	0.819
Dessert												
Day0	10.0 (0.0, 30.0)	0.016	1.6 (0.0, 4.4)	0.073	10.0 (0.0, 36.8)	0.260	0.7 (0.0, 4.5)	0.939			0.681	0.801
Day42	3.3 (0.0, 18.0)		0.6 (0.0, 3.6)		5.00 (0.0, 45.0)		0.3 (0.0, 3.8)				0.090	0.240
Change	−0.8 (−22.5, 0.0)		0.0 (−3.1, 1.1)		0.00 (−10.0, 2.5)		0.0 (0.0, 0.7)		−10.1 (−23.4 to 2.5)	−1.1 (−2.8 to 0.5)	0.114	0.192
Red meat												
Day0	50.0 (30.0, 150.0)	0.001	3.7 (1.7, 8.6)	0.006	75.0 (25.0, 150.0)	0.296	4.4 (1.7, 8.6)	0.683			0.325	0.321
Day42	30.0 (7.5, 60.0)		3.1 (0.5, 5.4)		60.0 (15.0, 150.0)		3.8 (1.3, 9.3)				0.009	0.035
Change	−17.5 (−57.5, 0.0)		−0.7 (−2.67, 0.0)		0.0 (−17.5, 5.0)		0.0 (−1.1, 1.2)		−35.8 (−79.2 to −6.5)	−1.7 (−3.7 to −0.4)	0.021	0.043
Poultry												
Day0	15.0 (0.0, 45.0)	0.030	0.9 (0.0, 2.7)	0.455	10.0 (0.0, 50.0)	0.319	0.8 (0.0, 3.1)	0.408			0.216	0.256
Day42	5.0 (0.0, 22.5)		0.4 (0.0, 2.4)		7.5 (0.0, 30.0)		0.6 (0.0, 2.0)				0.078	0.313
Change	0.0 (−17.5, 5.0)		0.0 (−1.1, 0.4)		0.0 (−7.5, 0.0)		0.0 (−0.6, 2.3)		−5.3 (−17.3 to 8.2)	−0.1 (−0.9 to 0.9)	0.484	0.901
Cereals												
Day0	343.8 ± 123.7	<0.001	46.1 (39.4, 55.1)	0.660	357.0 ± 120.8	0.264	45.3 (39.3, 54.0)	0.144			0.500	0.961
Day42	275.9 ± 87.7		48.5 (42.4, 54.5)		332.5 ± 95.2		49.5 (38.8, 58.2)				0.009	0.834
Change	−67.9 ± 117.2		0.0 (−6.9, 8.5)		−24.5 ± 117.2		1.1 (−0.4, 6.1)		−43.4 (−84.6 to 13.3)	−1.2 (−5.0 to 4.9)	0.154	0.800
Soy products												
Day0	8.0 (0.0, 22.5)	0.250	0.2 (0.0, 0.8)	0.208	5.0 (0.0, 30.0)	0.170	0.1 (0.0, 0.9)	0.362			0.970	0.606
Day42	2.5 (0.0, 18.0)		0.1 (0.0, 0.7)		2.5 (0.0, 10.0)		0.1 (0.0, 0.4)				0.324	0.529
Change	0.0 (−14.0, 0.0)		0.0 (−0.3, 0.1)		0.0 (−7.5, 0.0)		0.0 (−0.3, 0.0)		−10.9 (−39.9 to 20.9)	−0.3 (−1.1 to 0.4)	0.541	0.351
Mushrooms												
Day0	2.5 (0.0, 10.0)	0.089	0.0 (0.0, 0.2)	0.060	0.0 (0.0, 10.0)	0.806	0.0 (0.0, 0.1)	0.057			0.643	0.934
Day42	0.0 (0.0, 0.0)		0.0 (0.0, 0.0)		0.0 (0.0, 0.0)		0.0 (0.0, 0.0)				0.400	0.622
Change	0.0 (−10.0, 0.0)		0.0 (−0.1, 0.0)		0.0 (−7.5, 0.0)		0.0 (−0.1, 0.0)		−4.5 (−12.9 to 8.5)	0.0 (−0.1 to 0.1)	0.687	0.615
Fruits												
Day0	250.0 (150.0, 600.0)	0.047	5.4 (2.3, 11.7)	0.726	240.0 (120.0, 300.0)	0.580	4.2 (2.3, 7.0)	0.760			0.152	0.063
Day42	250.0 (120.0, 300.0)		5.3 (3.2, 8.0)		200.0 (75.0, 300.0)		3.4 (1.5, 7.9)				0.381	0.713
Change	0.0 (−10.0, 0.0)		0.0 (−3.7, 2.6)		0.0 (−80.0, 75.0)		0.0 (−1.6, 1.1)		−148.7 (−336.0 to 20.5)	−1.5 (−4.4 to −0.8)	0.027	0.165
Vegetables												
Day0	272.2 ± 121.8	<0.001	3.1 (1.8, 4.5)	<0.001	304.4 ± 148.8	0.121	2.8 (1.7, 5.0)	0.012			0.486	0.809
Day42	475.5 ± 154.0		7.0 (5.0, 8.7)		334.4 ± 139.3		4.1 (2.3, 5.7)				<0.001	<0.001
Change	203.3 ± 169.0		3.8 (1.9, 4.9)		30.0 ± 127.2		0.1 (−0.3, 1.4)		173.3 (91.5 to 224.4)	3.0 (1.9 to 3.7)	<0.001	<0.001
Eggs												
Day0	42.0 (0.0, 60.0)	<0.001	2.3 (0.0, 3.9)	<0.001	54.0 (0.0, 60.0)	0.788	2.1 (0.0, 4.1)	0.101			0.559	0.965
Day42	60.0 (60.0, 120.0)		4.8 (3.3, 8.4)		60.0 (0.0, 60.0)		2.7 (0.0, 4.2)				0.002	<0.001
Change	30.0 (0.0, 60.0)		2.8 (0.0, 5.1)		0.0 (0.0, 0.0)		0.0 (0.0, 0.5)		39.3 (21.7 to 60.0)	3.0 (1.8 to 4.2)	<0.001	<0.001
Dairy products												
Day0	75.0 (0.0, 250.0)	<0.001	2.0 (0.0, 4.4)	<0.001	25.0 (0.0, 210.0)	0.052	0.0 (0.0, 3.8)	0.074			0.212	0.219
Day42	250.0 (105.0, 250.0)		5.6 (2.2, 8.2)		150.0 (0.0, 250.0)		1.6 (0.0, 5.0)				0.002	0.001
Change	175.0 (0.0, 225.0)		2.0 (0.1, 6.4)		0.0 (0.0, 100.0)		0.0 (0.0, 1.7)		79.4 (1.2 to 163.4)	3.2 (1.1 to 5.5)	0.047	0.010
Oil												
Day0	49.1 ± 12.8	<0.001	17.9 (13.9, 23.6)	0.104	48.6 ± 12.1	0.004	16.4 (13.2, 19.8)	0.865			0.467	0.411
Day42	36.9 ± 7.0		17.2 (13.4, 9.5)		45.4 ± 10.7		16.7 (13.3, 19.5)				<0.001	0.751
Change	−12.2 ± 11.3		−2.0 (−4.5, 2.2)		−3.2 ± 7.24		0.0 (−1.4, 2.0)		−9.0 (−12.1 to −4.7)	−1.5 (−3.4 to 0.8)	<0.001	0.195

Food items, including shellfish, offal, broth, fish roe, sugar-sweetened beverages, and alcoholic beverages, were not presented in the baseline dietary intake table as over 75% of participants reported no consumption; however, their consumption data were included in the calculation of total nutrient intake. *p*-times for within-group changes (pre- vs. post-intervention) were calculated using paired *t*-tests. *p*-time ^1^ was expressed as the within-group difference in food weight. *p*-time ^2^ was expressed as the within-group difference in percentage of energy. *p*-values for between-group differences were derived from statistical models adjusted for potential confounding factors.

**Table 5:** Effects of LPEB diet on nutrient intake.

Time	Intervention Group (*n* = 45)				Control Group (*n* = 45)				Intervention Effect Estimate (95%CI)		*p*-Value	
	Nutrients	*p*-Time ^1^	% of Energy	*p*-Time ^2^	Nutrients	*p*-Time ^1^	% of Energy	*p*-Time ^2^	Nutrients	% of Energy	Foods	% of Energy
Energy, kcal/d												
Day0	2579.3 ± 702.4	<0.001		0.444	2755.1 ± 792.5	0.007		0.239			0.719	
Day42	2024.2 ± 378.0				2510.2 ± 607.8						<0.001	
Change	−555.0 ± 702.0				−244.9 ± 585.4				−310.1 (−580.9 to −39.4)		<0.001	
Carbohydrate, g/d												
Day0	361.2 ± 121.3	<0.001	55.6 ± 8.0	0.827	360.3 ± 110.8	0.202	52.8 ± 10.0	0.195			0.443	0.935
Day42	285.8 ± 75.9		55.8 ± 6.3		340.6 ± 91.2		54.6 ± 8.9				0.003	0.864
Change	−75.4 ± 115.0		0.2 ± 7.4		−19.6 ± 101.8		1.8 ± 9.4		−55.8 (−101.3 to −10.3)	−1.6 (−3.0 to 2.6)	0.003	0.891
Fat, g/d												
Day0	77.2 ± 17.5	<0.001	27.8 ± 6.3		80.9 ± 24.9	0.030	27.0 ± 5.8				0.478	0.276
Day42	62.8 ± 9.1		28.6 ± 5.2		76.9 ± 22.6		27.8 ± 5.8				<0.001	0.406
Change	−14.3 ± 17.3		0.7 ± 6.0		−4.0 ± 12.0		0.8 ± 4.5		−10.3 (−16.6 to −4.1)	−0.1 (−1.5 to 2.4)	<0.001	0.658
Protein, g/d												
Day0	94.3 ± 37.1	0.002	14.5 ± 3.6	0.084	108.0 ± 53.2	0.107	15.3 ± 4.0	0.048			0.949	0.696
Day42	77.4 ± 18.6		15.3 ± 2.5		102.0 ± 47.0		15.9 ± 4.5				0.002	0.458
Change	−16.8 ± 34.5		0.8 ± 3.0		−5.96 ± 24.31		0.6 ± 1.9		−10.9 (−23.4 to 1.6)	0.2 (−0.8 to 1.3)	0.002	0.599
Purine, mg/d												
Day0	644.2 ± 401.0	<0.001			798.8 ± 605.0	0.139					0.978	
Day42	399.2 ± 197.1				737.0 ± 540.8						<0.001	
Change	−245.0 ± 370.8				−61.8 ± 275.1				−183.1 (−319.9 to −46.4)		<0.001	

*p*-times for within-group changes (pre- vs. post-intervention) were calculated using paired *t*-tests. *p*-time ^1^ was expressed as the within-group difference in food weight. *p*-time ^2^ was expressed as the within-group difference in percentage of energy. *p*-values for between-group differences were derived from statistical models adjusted for potential confounding factors.

**Table 6:** Effects of LPEB diet on anthropometric parameters.

Time	Intervention Group (*n* = 45)	*p*-Time	Control Group (*n* = 45)	*p*-Time	Intervention Effect Estimate (95%CI)	*p*-Value
BMI, kg/m^2^						
Day0	27.4 ± 3.2	<0.001	26.8 ± 3.3	0.512		0.914
Day42	26.9 ± 3.1		26.9 ± 3.2			1.000
Change	−0.50 ± 0.65		0.05 ± 0.52		−0.55 (−0.75 to −0.25)	<0.001
Weight, kg						
Day0	83.3 ± 12.1	<0.001	80.6 ± 12.3	0.572		0.395
Day42	81.6 ± 11.9		80.8 ± 12.3			0.602
Change	−1.63 ± 2.01		0.14 ± 1.67		−1.77 (−2.35 to −0.78)	<0.001
SBP, mmHg						
Day0	134.6 ± 19.9	0.044	133.6 ± 15.2	0.220		0.943
Day42	131.0 ± 17.7		131.0 ± 13.4			0.956
Change	−3.56 ± 11.55		2.58 ± 13.85		−6.14 (−6.15 to 3.63)	0.447
DBP, mmHg						
Day0	85.9 ± 12.1	0.007	86.1 ± 10.0	0.138		0.302
Day42	82.6 ± 12.0		84.5 ± 10.3			0.156
Change	−3.31 ± 7.91		1.62 ± 7.21		−4.93 (−5.39 to 0.87)	0.213

*p*-times for within-group changes (pre- vs. post-intervention) were calculated using paired *t*-tests. *p*-values for between-group differences were derived from statistical models adjusted for potential confounding factors.

**Table 7:** Effects of LPEB diet on body composition parameters.

Time	Intervention Group (*n* = 45)	*p*-Time	Control Group (*n* = 45)	*p*-Time	Intervention Effect Estimate (95%CI)	*p*-Value
TBW, kg						
Day0	42.5 ± 6.5	0.295	41.7 ± 5.4	0.933		0.479
Day42	42.3 ± 6.4		41.7 ± 5.2			0.581
Change	−0.21 ± 1.31		−0.02 ± 1.76		−0.18 (−0.81 to 0.47)	0.604
Protein, kg						
Day0	11.4 ± 1.8	0.542	11.1 ± 1.5	0.954		0.505
Day42	11.4 ± 1.8		11.2 ± 1.4			0.574
Change	−0.03 ± 0.36		0.01 ± 0.52		−0.04 (−0.21 to 0.15)	0.724
Mineral, kg						
Day0	3.90 ± 0.66	0.543	3.77 ± 0.56	0.729		0.285
Day42	3.89 ± 0.65		3.76 ± 0.52			0.218
Change	−0.01 ± 0.16		−0.01 ± 0.23		0.00 (−0.67 to 0.09)	0.776
FAT, kg						
Day0	25.7 ± 5.6	<0.001	23.7 ± 7.6	0.155		0.143
Day42	23.3 ± 5.1		24.3 ± 7.3			0.174
Change	−2.39 ± 2.85		0.58 ± 2.68		−2.96 (−4.11 to −1.74)	<0.001
FFM, kg						
Day0	57.8 ± 8.9	0.339	56.6 ± 7.4	0.933		0.463
Day42	57.6 ± 8.8		56.6 ± 7.1			0.544
Change	−0.26 ± 1.82		−0.03 ± 2.48		−0.23 (−1.08 to 0.70)	0.666
SMM, kg						
Day0	32.4 ± 5.4	0.468	31.6 ± 4.5	0.961		0.487
Day42	32.3 ± 5.4		31.6 ± 4.3			0.552
Change	−0.12 ± 1.12		−0.01 ± 1.52		−0.11 (−0.64 to 0.45)	0.739
PBF, %						
Day0	30.7 ± 4.3	<0.001	29.0 ± 5.8	0.214		0.166
Day42	28.7 ± 4.6		29.5 ± 5.3			0.237
Change	−1.92 ± 2.94		0.58 ± 2.97		−2.48 (−3.73 to −1.23)	<0.001
BCM, kg						
Day0	37.8 ± 5.9	0.451	36.9 ± 4.9	0.979		0.392
Day42	37.6 ± 5.9		36.9 ± 4.8			0.468
Change	−0.14 ± 1.23		−0.01 ± 1.67		−0.13 (−0.72 to 0.48)	0.652
BMC, kg						
Day0	3.20 ± 0.56	0.677	3.09 ± 0.45	0.841		0.284
Day42	3.19 ± 0.55		3.08 ± 0.42			0.221
Change	−0.01 ± 0.14		−0.01 ± 0.19		0.00 (−0.06 to 0.07)	0.865
WC, cm						
Day0	97.6 ± 9.7	<0.001	94.9 ± 11.8	0.129		0.230
Day42	95.0 ± 8.9		95.9 ± 11.5			0.365
Change	−2.61 ± 3.90		1.03 ± 4.47		−3.64 (−5.33 to −1.68)	<0.001
VFA, cm^2^						
Day0	116.1 ± 28.5	<0.001	106.8 ± 39.5	0.147		0.169
Day42	104.0 ± 26.0		110.3 ± 37.9			0.199
Change	−12.1 ± 16.4		3.56 ± 16.2		−15.7(−22.6 to −8.7)	<0.001
BMR, kcal						
Day0	1619.0 ± 192.5	0.352	1592.2± 160.4	0.974		0.456
Day42	1613.6 ± 190.5		1592.4 ± 153.2			0.560
Change	−5.49 ± 39.10		0.27 ± 53.60		−5.75 (−24.67 to 14.05)	0.591

*p*-times for within-group changes (pre- vs. post-intervention) were calculated using paired *t*-tests. *p*-values for between-group differences were derived from statistical models adjusted for potential confounding factors. Abbreviation: TBW, total body water; FFM, fat-free mass; SMM, skeletal muscle mass; PBF, percent body fat; BCM, body cell mass; BMC, bone mineral content; WC, waist circumference; VFA, visceral fat area; BMR, basal metabolic rate.

**Table 8:** Effects of LPEB diet on biochemical parameters.

Time	Intervention Group (*n* = 45)	*p*-Time	Control Group (n = 45)	*p*-Time	Intervention Effect Estimate (95%CI)	*p*-Value
ALT, U/L						
Day0	35.9 ± 20.8	0.009	40.6 ± 24.4	0.118		0.543
Day42	31.4 ± 17.2		37.1 ± 20.2			0.388
Change	−4.49 ± 10.92		−3.44 ± 14.53		−1.04 (−6.06 to 4.92)	0.839
AST, U/L						
Day0	26.0 ± 12.1	0.259	26.3 ± 9.8	0.152		0.880
Day42	24.7 ± 8.91		24.3 ± 7.72			0.471
Change	−1.38 ± 8.08		−1.98 ± 9.10		0.60 (−2.63 to 4.59)	0.594
TG, mmol/L						
Day0	2.23 ± 1.05	<0.001	1.92 ± 1.03	0.331		0.314
Day42	1.83 ± 0.93		2.08 ± 1.27			0.186
Change	−0.40 ± 0.52		0.17 ± 1.12		−0.51 (−1.06 to −0.08)	0.023
TC, mmol/L						
Day0	5.12 ± 1.09	0.105	4.93 ± 1.02	0.155		0.326
Day42	4.90 ± 0.91		4.73 ± 1.01			0.470
Change	−0.22 ± 0.89		−0.20 ± 0.95		−0.02 (−0.46 to 0.32)	0.722
BG, mmol/L						
Day0	5.79 ± 0.92	0.075	5.55 ± 0.98	0.656		0.614
Day42	5.64 ± 1.01		5.60 ± 0.81			0.732
Change	−0.15 ± 0.54		0.05 ± 0.72		−0.19 (−0.44 to 0.12)	0.254
UA, µmol/L						
Day0	470.6 ± 102.3	<0.001	466.6 ± 87.1	0.001		0.570
Day42	358.2 ± 111.8		419.6 ± 126.2			0.036
Change	−112.4 ± 103.7		−47.0 ± 116.9		−65.4 (−116.1 to −18.2)	0.007
BUN, mmol/L						
Day0	4.49 ± 1.52	0.020	5.08 ± 1.21	0.070		0.002
Day42	4.94 ± 1.21		4.79 ± 1.12			0.804
Change	0.45 ± 1.24		−0.29 ± 1.01		0.77 (0.29 to 1.23)	0.002
CREA, µmol/L						
Day0	85.2 ± 17.2	0.015	81.9 ± 16.7	0.413		0.737
Day42	81.4 ± 14.8		83.1 ± 17.1			0.479
Change	−3.84 ± 10.2		1.16 ± 9.39		−5.00 (−9.05 to −0.95)	0.016
GFR, mL/min/1.73 m^2^						
Day0	96.0 ± 22.5	0.019	96.8 ± 22.5	0.282		0.978
Day42	100.7 ± 24.3		94.8 ± 20.2			0.204
Change	4.67 ± 12.8		−1.98 ± 12.2		5.92 (−0.25 to −8.26)	0.038
CREA-U, mmol/L						
Day0	10.4 ± 8.3	0.018	11.6 ± 6.1	0.904		0.309
Day42	13.5 ± 7.4		11.7 ± 5.5			0.394
Change	3.05 ± 8.30		0.10 ± 5.78		2.95 (−0.12 to 5.96)	0.060
UA-U, mmol/L						
Day0	1.99 ± 1.29	0.052	2.22 ± 1.23	0.937		0.228
Day42	2.37 ± 1.20		2.23 ± 1.20			0.956
Change	0.37 ± 1.25		0.01 ± 0.99		0.36 (−0.18 to 0.83)	0.209
FEUA, %						
Day0	3.98 ± 2.16	0.002	4.24 ± 1.82	0.292		0.226
Day42	4.85 ± 3.87		4.02 ± 1.38			0.126
Change	0.87 ± 2.60		−0.21 ± 1.39		1.1 (0.51 to 1.92)	0.001
pH-U						
Day0	5.82 ± 0.64	0.874	5.93 ± 0.70	0.495		0.441
Day42	5.81 ± 0.63		5.88 ± 0.65			0.919
Change	−0.01 ± 0.48		−0.05 ± 0.46		0.07 (−0.35 to 0.49)	0.494

*p*-values for within-group changes (pre- vs. post-intervention) were calculated using paired *t*-tests. *p*-values for between-group differences were derived from statistical models adjusted for potential confounding factors. Abbreviation: ALT, alanine aminotransferase; AST, aspartate aminotransferase; TC, total cholesterol; TG, triglycerides; BG, blood glucose; UA, uric acid; BUN, blood urea nitrogen; CREA, creatinine; GFR, glomerular filtration rate; CREA-U, urinary creatinine; UA-U, urinary uric acid; FEUA, fraction excretion of uric acid, calculated as (urinary uric acid × serum creatinine)/(serum uric acid × urinary creatinine) × 100%; pH-U, urine pH.

## Data Availability

The raw data supporting the findings of this study have been submitted to the ScienceDB repository and are currently undergoing review for public release. Upon completion of the review process, the data will be openly accessible via the permanent link: https://www.scidb.cn/s/ARbqEr (15 May 2026) and the pre-allocated DOI: https://doi.org/10.57760/sciencedb.37304. For access during the peer-review process, please contact the corresponding author.

## Supplementary Materials

The following supporting information can be downloaded at: https://www.mdpi.com/article/10.3390/nu18132047/s1. File S1 CONSORT2025 Checklist. Table S1A. Example of a Low-Purine Diet for 1400 kcal (Purine Intake < 200 mg) Table S1B. Example of a Low-Purine Diet for 1600 kcal (Purine Intake < 200 mg) Table S1C. Example of a Low-Purine Diet for 1800 kcal (Purine Intake < 200 mg) Table S1D. Example of a Low-Purine Diet for 2000 kcal (Purine Intake < 200 mg) Table S1E. Example of a Low-Purine Diet for 1400 kcal (Purine Intake < 600 mg) Table S1F. Example of a Low-Purine Diet for 1600 kcal (Purine Intake < 600 mg) Table S1G. Example of a Low-Purine Diet for 1800 kcal (Purine Intake < 600 mg) Table S1H. Example of a Low-Purine Diet for 2000 kcal (Purine Intake < 600 mg) Table S2. Effects of an LPEB Diet on Serum Biochemical Parameters: Per Protocol Analysis.

## Abbreviations

The following abbreviations are used in this manuscript:

LPEB diet	low-purine, energy-restricted and balanced diet
sUA	serum uric acid
FEUA	fractional excretion of uric acid
VFA	visceral fat area
