# Supplementary material for: Efficacy of a Low-Purine, Energy-Restricted and Balanced Diet on Hyperuricemia and Metabolic Profiles in Gout Patients: A Randomized Controlled Trial
Source: Nutrients. 2026 Jun 23;18(13):2047. doi: 10.3390/nu18132047 (PMC13363501; doi:10.3390/nu18132047)
Supplement: Supplementary file 1 [file nutrients-18-02047-s001.zip › Supplemental tables.pdf]

Supplemental Table S1A. Example of a Low-Purine Diet for 1400 kcal (Purine Intake <200mg)

DAY	Breakfast		Lunch		Snack		Dinner	
	**Foods**	**Quantity**	**Foods**	**Quantity**	**Foods**	**Quantity**	**Foods**	**Quantity**
DAY1	Bread	2 slice (Flour 50g)	Steamed Bread	105g (Flour 75g)	Apple	150g	Steamed Bread	75g (Flour50g)
	Egg	60g	Braised Lean Pork with Chinese Cabbage	Chinase Cabbage 250g<br>Pork 25g			Saute Tomato with Egg	Tomato 250g<br>Egg 60g
	Skim Milk	250ml	Skim Milk	250ml				
			Oil	15g			Oil	10g
DAY2	Noodles	1 bowl (Flour 50g)<br>Cucumber 50g	Steamed Rice	165g (Rice 75g)	Cherry	150g	Steamed Bread	75g (Flour50g)
			Saute Beef Tenderloin with Green Pepper	Green Pepper 250g<br>Beef Tenderloin 25g			White Gourd Egg Soup	White Gourd 250g<br>Egg 60g
	Egg	60g						
	Skim Milk	250ml	Skim Milk	250ml				
			Oil	15g			Oil	10g
DAY3	Steamed Bread	70g (Flour 50g)	Steamed Bread	105g (Flour 75g)	Pear	150g	Steamed Bread	75g (Flour50g)
	Bitter Melon in Sauce	Bitter Melon 50g	Saute Chinesecelery Cabbage with Pork	Chinesecelery Cabbage 250g<br>Pork 25g			Saute Zucchini with Egg	Zucchini 250g<br>Egg 60g
	Egg	60g						
	Skim Milk	250ml	Skim Milk	250ml				
			Oil	15g			Oil	10g
DAY4	Steamed Bread	70g (Flour 50g)	Steamed Bread	105g (Flour 75g)	Peach	150g	Steamed Rice	110g (Rice 50g)
	Celery Salad	Celery 50g	Saute Spinage with Chicken	Spinage 250g<br>Chicken 25g			Saute Towel Gourd with Egg	Towel Gourd 250g<br>Egg 60g
	Egg	60g						
	Skim Milk	250ml	Skim Milk	250ml				
			Oil	15g			Oil	10g

DAY5	Steamed Yam	Yam100g	Steamed Bread	105g (Flour 75g)	Watermelon	150g	Steamed Rice	110g (Rice 50g)
	Steamed Bread	35g(Flour 25g)	Saute Cabbage with Pork	Cabbage 250g			Saute Spinage with Egg	Spinage 250g
	Egg	60g		Pork 25g				Egg 60g
	Skim Milk	250ml	Skim Milk	250ml				
			Oil	15g			Oil	10g
DAY6	Bread	1 slice (Flour 50g)	Steamed Rice	165g (Rice 75g)	Strawberry	150g	Steamed Rice	110g (Rice 50g)
	Egg	60g	Saute Lettuce with Pork	Lettuce 250g			Saute Cucumber with Egg	Cucumber 250g
	Skim Milk	250ml		Pork 25g				Egg 60g
			Skim Milk	250ml				
			Oil	15g			Oil	10g
DAY7	Noodles	1 bowl (Flour 50g)	Steamed Rice	165g (Rice 75g)	Watermelon	150g	Steamed Rice	110g (Rice 50g)
		Cucumber 50g	Saute Eggplant with Pork	Eggplant 250g			Saute Cabbage with Egg	Cabbage 250g
	Egg	60g		Pork 25g				Egg 60g
	Skim Milk	250ml	Skim Milk	250ml				
			Oil	15g			Oil	10g

Supplemental Table S1B. Example of a Low-Purine Diet for 1600 kcal (Purine Intake < 200mg)

DAY	Breakfast		Lunch		Snack		Dinner	
	Foods	Quantity	Foods	Quantity	Foods	Quantity	Foods	Quantity
DAY1	Bread	2 slice (Flour 50g)	Steamed Bread	105g (Flour 75g)	Apple	150g	Steamed Bread	105g (Flour 75g)
	Egg	60g	Braised Lean Pork with Chinese Cabbage	Chinase Cabbage 250g			Saute Tomato with Egg	Tomato 250g
	Skim Milk	250ml		Pork 25g				Egg 120g
			Skim Milk	250ml				
			Oil	15g			Oil	15g
DAY2	Noodles	1 bowl (Flour 50g)	Steamed Rice	165g (Rice 75g)	Cherry	150g	Steamed Bread	105g (Flour 75g)
		Cucumber 50g	Saute Beef Tenderloin with Green Pepper	Green Pepper 250g			White Gourd Egg Soup	White Gourd 250g
	Egg	60g		Beef Tenderloin 25g				Egg 120g
	Skim Milk	250ml	Skim Milk	250ml				
			Oil	15g			Oil	15g
DAY3	Steamed Bread	75g (Flour50g)	Steamed Bread	105g (Flour 75g)	Pear	150g	Steamed Bread	105g (Flour 75g)
	Bitter Melon in Sauce	Bitter Melon 50g	Saute Chinesecelery Cabbage with Pork	Chinesecelery Cabbage 250g			Saute Zucchini with Egg	Zucchini 250g
	Egg	60g		Pork 25g				Egg 120g
	Skim Milk	250ml	Skim Milk	250ml				
			Oil	15g			Oil	15g
DAY4	Steamed Bread	75g (Flour50g)	Steamed Bread	105g (Flour 75g)	Peach	150g	Steamed Rice	165g (Rice 75g)
	Celery Salad	Celery 50g	Saute Spinage with Chicken	Spinage 250g			Saute Towel Gourd with Egg	Towel Gourd 250g
	Egg	60g		Chicken 25g				Egg 120g
	Skim Milk	250ml	Skim Milk	250ml				

			Oil	15g			Oil	15g
DAY5	Steamed Yam	Yam 100g	Steamed Bread	105g (Flour 75g)	Watermelon	150g	Steamed Rice	165g (Rice 75g)
	Steamed Bread	35g (Flour 25g)	Saute Cabbage with Pork	Cabbage 250g			Saute Spinage with Egg	Spinage 250g
	Egg	60g		Pork 25g				Egg 120g
	Skim Milk	250ml	Skim Milk	250ml				
			Oil	15g			Oil	15g
DAY6	Bread	2 slice (Flour 50g)	Steamed Rice	165g (Rice 75g)	Strawberry	150g	Steamed Rice	165g (Rice 75g)
	Egg	60g	Saute Lettuce with Pork	Lettuce 250g			Saute Cucumber with Egg	Cucumber 250g
	Skim Milk	250ml		Pork 25g				Egg 120g
			Skim Milk	250ml				
			Oil	15g			Oil	15g
DAY7	Noodles	1 bowl (Flour 50g)	Steamed Rice	165g (Rice 75g)	Watermelon	150g	Steamed Rice	165g (Rice 75g)
		Cucumber 50g	Saute Eggplant with Pork	Eggplant 250g			Saute Cabbage with Egg	Cabbage 250g
	Egg	60g		Pork 25g				Egg 120g
	Skim Milk	250ml	Skim Milk	250ml				
			Oil	15g			Oil	15g

Supplemental Table S1C. Example of a Low-Purine Diet for 1800 kcal (Purine Intake < 200mg)

DAY	Breakfast		Lunch		Snack		Dinner	
	Foods	Quantity	Foods	Quantity	Foods	Quantity	Foods	Quantity
DAY1	Bread	2 slice (Flour 50g)	Steamed Bread	140g (Flour 100g)	Apple	150g	Steamed Bread	105g (Flour 75g)
	Egg	60g	Braised Lean Pork with Chinese Cabbage	Chinase Cabbage 250g	Skim Milk	250ml	Saute Tomato with Egg	Tomato 250g
	Skim Milk	250ml		Pork 25g				Egg 120g
			Saute Cabbage	Cabbage 100g				
			Skim Milk	250ml				
			Oil	15g			Oil	15g
DAY2	Noodles	1 bowl (Flour 50g)	Steamed Rice	220g (Rice 100g)	Cherry	150g	Steamed Bread	105g (Flour 75g)
		Cucumber 50g	Saute Beef Tenderloin with Green Pepper	Green Pepper 250g	Skim Milk	250ml	White Gourd Egg Soup	White Gourd 250g
	Egg	60g		Beef Tenderloin 25g				Egg 120g
	Skim Milk	250ml	Saute Chinesecelery Cabbage	Chinesecelery Cabbage 100g				
			Skim Milk	250ml				
			Oil	15g			Oil	15g
DAY3	Steamed Bread	75g (Flour50g)	Steamed Bread	140g (Flour 100g)	Pear	150g	Steamed Bread	105g (Flour 75g)
	Bitter Melon in Sauce	Bitter Melon 50g	Saute Chinesecelery Cabbage with Pork	Chinesecelery Cabbage 250g	Skim Milk	250ml	Saute Zucchini with Egg	Zucchini 250g
	Egg	60g		Pork 25g				Egg 120g
	Skim Milk	250ml	Saute Radish	Radish 100g				
			Skim Milk	250ml				
			Oil	15g			Oil	15g
DAY4	Steamed Bread	75g (Flour50g)	Steamed Bread	140g (Flour 100g)	Peach	150g	Steamed Rice	165g (Rice 75g)
	Celery Salad	Celery 50g	Saute Spinage with	Spinage 250g	Skim Milk	250ml	Saute Towel	Towel Gourd 250g

	Egg	60g	Chicken	Chicken 25g			Gourd with Egg	Egg 120g
	Skim Milk	250ml	Saute Mungbean sprout	Mungbean sprout 100g				
			Skim Milk	250ml				
			Oil	15g			Oil	15g
DAY5	Steamed Yam	Yam 100g	Steamed Bread	140g (Flour 100g)	Watermelon	150g	Steamed Rice	165g (Rice 75g)
	Steamed Bread	35g (Flour 25g)	Saute Cabbage with Pork	Cabbage 250g	Skim Milk	250ml	Saute Spinage with Egg	Spinage 250g
	Egg	60g		Pork 25g				Egg 120g
	Skim Milk	250ml	Saute Celery	Celery 100g				
			Skim Milk	250ml				
			Oil	15g			Oil	15g
DAY6	Bread	2 slice (Flour 50g)	Steamed Rice	220g (Rice 100g)	Strawberry	150g	Steamed Rice	165g (Rice 75g)
	Egg	60g	Saute Lettuce with Pork	Lettuce 250g	Skim Milk	250ml	Saute Cucumber with Egg	Cucumber 250g
	Skim Milk	250ml		Pork 25g				Egg 120g
			Saute Cabbage	Cabbage 100g				
			Skim Milk	250ml				
			Oil	15g			Oil	15g
DAY7	Noodles	1 bowl (Flour 50g)	Steamed Rice	220g (Rice 100g)	Watermelon	150g	Steamed Rice	165g (Rice 75g)
		Cucumber 50g	Saute Eggplant with Pork	Eggplant 250g	Skim Milk	250ml	Saute Cabbage with Egg	Cabbage 250g
	Egg	60g		Pork 25g				Egg 120g
	Skim Milk	250ml	Saute Celery	Celery 100g				
			Skim Milk	250ml				
			Oil	15g			Oil	15g

Supplemental Table S1D. Example of a Low-Purine Diet for 2000 kcal (Purine Intake < 200mg)

DAY	Breakfast		Lunch		Snack		Dinner	
	Foods	Quantity	Foods	Quantity	Foods	Quantity	Foods	Quantity
DAY1	Bread	3 slice (Flour 75g)	Steamed Bread	170g (Flour 125g)	Apple	150g	Steamed Bread	105g (Flour 75g)
	Egg	60g	Braised Lean Pork with Chinese Cabbage	Chinase Cabbage 200g	Skim Milk	250ml	Saute Tomato with Egg	Tomato 250g
	Skim Milk	250ml		Pork 25g				Egg 120g
			Saute Cabbage	Cabbage 200g				
			Skim Milk	250ml				
			Oil	15g			Oil	15g
DAY2	Noodles	1 bowl (Flour 75g)	Steamed Rice	275g (Rice 125g)	Cherry	150g	Steamed Bread	105g (Flour 75g)
		Cucumber 50g	Saute Beef Tenderloin with Green Pepper	Green Pepper 200g	Skim Milk	250ml	White Gourd Egg Soup	White Gourd 250g
	Egg	60g		Beef Tenderloin 25g				Egg 120g
	Skim Skim Milk	250ml	Saute Chinesecelery Cabbage	Chinesecelery Cabbage 200g				
			Skim Milk	250ml				
			Oil	15g			Oil	15g
DAY3	Steamed Bread	105g (Flour 75g)	Steamed Bread	170g (Flour 125g)	Pear	150g	Steamed Bread	105g (Flour 75g)
	Bitter Melon in Sauce	Bitter Melon 50g	Saute Chinesecelery Cabbage with Pork	Chinesecelery Cabbage 200g	Skim Milk	250ml	Saute Zucchini with Egg	Zucchini 250g
	Egg	60g		Pork 25g				Egg 120g
	Skim Skim Milk	250ml	Saute Radish	Radish 200g				
			Skim Milk	250ml				
			Oil	15g			Oil	15g
DAY4	Steamed Bread	105g (Flour 75g)	Steamed Bread	170g (Flour 125g)	Peach	150g	Steamed Rice	165g (Rice 75g)
	Celery Salad	Celery 50g	Saute Spinage with	Spinage 200g	Skim Milk	250ml	Saute Towel	Towel Gourd 250g

	Egg	60g	Chicken	Chicken 25g			Gourd with Egg	Egg 120g
	Skim Skim Milk	250ml	Saute Mungbean sprout	Mungbean sprout 200g				
			Skim Milk	250ml				
			Oil	15g			Oil	15g
DAY5	Steamed Yam	Yam 100g	Steamed Bread	170g (Flour 125g)	Watermelon	150g	Steamed Rice	165g (Rice 75g)
	Steamed Bread	75g (Flour 50g)	Saute Cabbage with Pork	Cabbage 200g	Skim Milk	250ml	Saute Spinage with Egg	Spinage 250g
	Egg	60g		Pork 25g				Egg 120g
	Skim Skim Milk	250ml	Saute Celery	Celery 200g				
			Skim Milk	250ml				
			Oil	15g			Oil	15g
DAY6	Bread	3 slice (Flour 75g)	Steamed Rice	275g (Rice 125g)	Strawberry	150g	Steamed Rice	165g (Rice 75g)
	Egg	60g	Saute Lettuce with Pork	Lettuce 200g	Skim Milk	250ml	Saute Cucumber with Egg	Cucumber 250g
	Skim Skim Milk	250ml		Pork 25g				Egg 120g
			Saute Cabbage	Cabbage 200g				
			Skim Milk	250ml				
			Oil	15g			Oil	15g
DAY7	Noodles	1 bowl (Flour 70g)	Steamed Rice	275g (Rice 125g)	Watermelon	150g	Steamed Rice	165g (Rice 75g)
		Cucumber 50g	Saute Eggplant with Pork	Eggplant 200g	Skim Milk	250ml	Saute Cabbage with Egg	Cabbage 250g
	Egg	60g		Pork 25g				Egg 120g
	Skim Skim Milk	250ml	Saute Celery	Celery 200g				
			Skim Milk	250ml				
			Oil	15g			Oil	15g

Supplemental Table S1E. Example of a Low-Purine Diet for 1400 kcal (Purine Intake < 600mg)

DAY	Breakfast		Lunch		Snack		Dinner	
	Foods	Quantity	Foods	Quantity	Foods	Quantity	Foods	Quantity
DAY1	Bread	1 slice (Flour 25g)	Steamed Bread	105g (Flour 75g)	Apple	150g	Steamed Bread	75g (Flour50g)
	Egg	60g	Braised Lean Pork with Chinese Cabbage	Chinase Cabbage 250g			Saute Tomato with Egg	Tomato 250g
	Skim Milk	250ml		Pork 75g				Egg 60g
			Skim Milk	250ml				
			Oil	15g			Oil	10g
DAY2	Noodles	1 bowl (Flour 25g)	Steamed Rice	165g (Rice 75g)	Cherry	150g	Steamed Bread	75g (Flour50g)
		Cucumber 50g	Saute Beef Tenderloin with Green Pepper	Green Pepper 250g			White Gourd Egg Soup	White Gourd 250g
	Egg	60g		Beef Tenderloin 75g				Egg 60g
	Skim Milk	250ml	Skim Milk	250ml				
			Oil	15g			Oil	10g
DAY3	Steamed Bread	35g (Flour 25g)	Steamed Bread	105g (Flour 75g)	Pear	150g	Steamed Bread	75g (Flour50g)
	Bitter Melon in Sauce	Bitter Melon 50g	Saute Chinesecelery Cabbage with Pork	Chinesecelery Cabbage 250g			Saute Zucchini with Egg	Zucchini 250g
	Egg	60g		Pork 75g				Egg 60g
	Skim Milk	250ml	Skim Milk	250ml				
			Oil	15g			Oil	10g
DAY4	Steamed Bread	35g (Flour 25g)	Steamed Bread	105g (Flour 75g)	Peach	150g	Steamed Rice	110g (Rice 50g)
	Celery Salad	Celery 50g	Saute Spinage with Chicken	Spinage 250g			Saute Towel Gourd with Egg	Towel Gourd 250g
	Egg	60g		Chicken 75g				Egg 60g
	Skim Milk	250ml	Skim Milk	250ml				

			Oil	15g			Oil	10g
DAY5	Steamed Yam	Yam100g	Steamed Bread	105g (Flour 75g)	Watermelon	150g	Steamed Rice	110g (Rice 50g)
	Egg	60g	Saute Cabbage with Pork	Cabbage 250g			Saute Spinage with Egg	Spinage 250g
	Skim Milk	250ml		Pork 75g				Egg 60g
			Skim Milk	250ml				
			Oil	15g			Oil	10g
DAY6	Bread	1 slice (Flour 25g)	Steamed Rice	165g (Rice 75g)	Strawberry	150g	Steamed Rice	110g (Rice 50g)
	Egg	60g	Saute Lettuce with Pork	Lettuce 250g			Saute Cucumber with Egg	Cucumber 250g
	Skim Milk	250ml		Pork 75g				Egg 60g
			Skim Milk	250ml				
			Oil	15g			Oil	10g
DAY7	Noodles	1 bowl (Flour 25g)	Steamed Rice	165g (Rice 75g)	Watermelon	150g	Steamed Rice	110g (Rice 50g)
		Cucumber 50g	Saute Eggplant with Pork	Eggplant 250g			Saute Cabbage with Egg	Cabbage 250g
	Egg	60g		Pork 75g				Egg 60g
	Skim Milk	250ml	Skim Milk	250ml				
			Oil	15g			Oil	10g

Supplemental Table S1F. Example of a Low-Purine Diet for 1600 kcal (Purine Intake < 600mg)

DAY	Breakfast		Lunch		Snack		Dinner	
	Foods	Quantity	Foods	Quantity	Foods	Quantity	Foods	Quantity
DAY1	Bread	2 slice (Flour 50g)	Steamed Bread	105g (Flour 75g)	Apple	150g	Steamed Bread	75g (Flour50g)
	Egg	60g	Braised Lean Pork with Chinese Cabbage	Chinase Cabbage 250g			Saute Tomato with Egg	Tomato 250g
	Skim Milk	250ml		Pork 75g				Egg 60g
			Skim Milk	250ml				
			Oil	15g			Oil	15g
DAY2	Noodles	1 bowl (Flour 50g)	Steamed Rice	165g (Rice 75g)	Cherry	150g	Steamed Bread	75g (Flour50g)
		Cucumber 50g	Saute Beef Tenderloin with Green Pepper	Green Pepper 250g			White Gourd Egg Soup	White Gourd 250g
	Egg	60g		Beef Tenderloin 75g				Egg 60g
	Skim Milk	250ml	Skim Milk	250ml				
			Oil	15g			Oil	15g
DAY3	Steamed Bread	75g (Flour50g)	Steamed Bread	105g (Flour 75g)	Pear	150g	Steamed Bread	75g (Flour50g)
	Bitter Melon in Sauce	Bitter Melon 50g	Saute Chinesecelery Cabbage with Pork	Chinesecelery Cabbage 250g			Saute Zucchini with Egg	Zucchini 250g
	Egg	60g		Pork 75g				Egg 60g
	Skim Milk	250ml	Skim Milk	250ml				
			Oil	15g			Oil	15g
DAY4	Steamed Bread	75g (Flour50g)	Steamed Bread	105g (Flour 75g)	Peach	150g	Steamed Rice	110g (Rice 50g)
	Celery Salad	Celery 50g	Saute Spinage with Chicken	Spinage 250g			Saute Towel Gourd with Egg	Towel Gourd 250g
	Egg	60g		Chicken 75g				Egg 60g
	Skim Milk	250ml	Skim Milk	250ml				
			Oil	15g			Oil	15g

DAY5	Steamed Yam	Yam 100g	Steamed Bread	105g (Flour 75g)	Watermelon	150g	Steamed Rice	110g (Rice 50g)
	Steamed Bread	35g (Flour 25g)	Saute Cabbage with Pork	Cabbage 250g			Saute Spinage with Egg	Spinage 250g
	Egg	60g		Pork 75g				Egg 60g
	Skim Milk	250ml	Skim Milk	250ml				
			Oil	15g			Oil	15g
DAY6	Bread	2 slice (Flour 50g)	Steamed Rice	165g (Rice 75g)	Strawberry	150g	Steamed Rice	110g (Rice 50g)
	Egg	60g	Saute Lettuce with Pork	Lettuce 250g			Saute Cucumber with Egg	Cucumber 250g
	Skim Milk	250ml		Pork 75g				Egg 60g
			Skim Milk	250ml				
			Oil	15g			Oil	15g
DAY7	Noodles	1 bowl (Flour 50g)	Steamed Rice	165g (Rice 75g)	Watermelon	150g	Steamed Rice	110g (Rice 50g)
		Cucumber 50g	Saute Eggplant with Pork	Eggplant 250g			Saute Cabbage with Egg	Cabbage 250g
	Egg	60g		Pork 75g				Egg 60g
	Skim Milk	250ml	Skim Milk	250ml				
			Oil	15g			Oil	15g

Supplemental Table S1G. Example of a Low-Purine Diet for 1800 kcal (Purine Intake < 600mg)

DAY	Breakfast		Lunch		Snack		Dinner	
	Foods	Quantity	Foods	Quantity	Foods	Quantity	Foods	Quantity
DAY1	Bread	2 slice (Flour 50g)	Steamed Bread	105g (Flour 75g)	Apple	150g	Steamed Bread	105g (Flour 75g)
	Egg	60g	Braised Lean Pork with Chinese Cabbage	Chinase Cabbage 250g	Skim Milk	250ml	Saute Tomato with Egg	Tomato 250g
	Skim Milk	250ml		Pork 75g				Egg 120g
			Saute Cabbage	Cabbage 100g				
			Skim Milk	250ml				
			Oil	15g			Oil	15g
DAY2	Noodles	1 bowl (Flour 50g)	Steamed Rice	165g (Rice 75g)	Cherry	150g	Steamed Bread	105g (Flour 75g)
		Cucumber 50g	Saute Beef Tenderloin with Green Pepper	Green Pepper 250g	Skim Milk	250ml	White Gourd Egg Soup	White Gourd 250g
	Egg	60g		Beef Tenderloin 75g				Egg 120g
	Skim Milk	250ml	Saute Chinesecelery Cabbage	Chinesecelery Cabbage 100g				
			Skim Milk	250ml				
			Oil	15g			Oil	15g
DAY3	Steamed Bread	75g (Flour50g)	Steamed Bread	105g (Flour 75g)	Pear	150g	Steamed Bread	105g (Flour 75g)
	Bitter Melon in Sauce	Bitter Melon 50g	Saute Chinesecelery Cabbage with Pork	Chinesecelery Cabbage 250g	Skim Milk	250ml	Saute Zucchini with Egg	Zucchini 250g
	Egg	60g		Pork 75g				Egg 120g
	Skim Milk	250ml	Saute Radish	Radish 100g				
			Skim Milk	250ml				
			Oil	15g			Oil	15g
DAY4	Steamed Bread	75g (Flour50g)	Steamed Bread	105g (Flour 75g)	Peach	150g	Steamed Rice	165g (Rice 75g)
	Celery Salad	Celery 50g	Saute Spinage with	Spinage 250g	Skim Milk	250ml	Saute Towel	Towel Gourd 250g

	Egg	60g	Chicken	Chicken 75g			Gourd with Egg	Egg 120g
	Skim Milk	250ml	Saute Mungbean sprout	Mungbean sprout 100g				
			Skim Milk	250ml				
			Oil	15g			Oil	15g
DAY5	Steamed Yam	Yam 100g	Steamed Bread	105g (Flour 75g)	Watermelon	150g	Steamed Rice	165g (Rice 75g)
	Steamed Bread	35g(Flour 25g)	Saute Cabbage with Pork	Cabbage 250g	Skim Milk	250ml	Saute Spinage with Egg	Spinage 250g
	Egg	60g		Pork 75g				Egg 120g
	Skim Milk	250ml	Saute Celery	Celery 100g				
			Skim Milk	250ml				
			Oil	15g			Oil	15g
DAY6	Bread	2 slice (Flour 50g)	Steamed Rice	165g (Rice 75g)	Strawberry	150g	Steamed Rice	165g (Rice 75g)
	Egg	60g	Saute Lettuce with Pork	Lettuce 250g	Skim Milk	250ml	Saute Cucumber with Egg	Cucumber 250g
	Skim Milk	250ml		Pork 75g				Egg 120g
			Saute Cabbage	Cabbage 100g				
			Skim Milk	250ml				
			Oil	15g			Oil	15g
DAY7	Noodles	1 bowl (Flour 50g)	Steamed Rice	165g (Rice 75g)	Watermelon	150g	Steamed Rice	165g (Rice 75g)
		Cucumber 50g	Saute Eggplant with Pork	Eggplant 250g	Skim Milk	250ml	Saute Cabbage with Egg	Cabbage 250g
	Egg	60g		Pork 75g				Egg 120g
	Skim Milk	250ml	Saute Celery	Celery 100g				
			Skim Milk	250ml				
			Oil	15g			Oil	15g

Supplemental Table S1H. Example of a Low-Purine Diet for 2000 kcal (Purine Intake < 600mg)

DAY	Breakfast		Lunch		Snack		Dinner	
	Foods	Quantity	Foods	Quantity	Foods	Quantity	Foods	Quantity
DAY1	Bread	3 slice (Flour 75g)	Steamed Bread	140g (Flour 100g)	Apple	150g	Steamed Bread	105g (Flour 75g)
	Egg	60g	Braised Lean Pork with Chinese Cabbage	Chinase Cabbage 200g	Skim Milk	250ml	Saute Tomato with Egg	Tomato 250g
	Skim Milk	250ml		Pork 75g				Egg 120g
			Saute Cabbage	Cabbage 200g				
			Skim Milk	250ml				
			Oil	15g			Oil	15g
DAY2	Noodles	1 bowl (Flour 75g)	Steamed Rice	220g (Rice 100g)	Cherry	150g	Steamed Bread	105g (Flour 75g)
		Cucumber 50g	Saute Beef Tenderloin with Green Pepper	Green Pepper 200g	Skim Milk	250ml	White Gourd Egg Soup	White Gourd 250g
	Egg	60g		Beef Tenderloin 75g				Egg 120g
	Skim Skim Milk	250ml	Saute Chinesecelery Cabbage	Chinesecelery Cabbage 200g				
			Skim Milk	250ml				
			Oil	15g			Oil	15g
DAY3	Steamed Bread	105g (Flour 75g)	Steamed Bread	140g (Flour 100g)	Pear	150g	Steamed Bread	105g (Flour 75g)
	Bitter Melon in Sauce	Bitter Melon 50g	Saute Chinesecelery Cabbage with Pork	Chinesecelery Cabbage 200g	Skim Milk	250ml	Saute Zucchini with Egg	Zucchini 250g
	Egg	60g		Pork 75g				Egg 120g
	Skim Skim Milk	250ml	Saute Radish	Radish 200g				
			Skim Milk	250ml				
			Oil	15g			Oil	15g
DAY4	Steamed Bread	105g (Flour 75g)	Steamed Bread	140g (Flour 100g)	Peach	150g	Steamed Rice	165g (Rice 75g)
	Celery Salad	Celery 50g	Saute Spinage with	Spinage 200g	Skim Milk	250ml	Saute Towel	Towel Gourd 250g

	Egg	60g	Chicken	Chicken 75g			Gourd with Egg	Egg 120g
	Skim Skim Milk	250ml	Saute Mungbean sprout	Mungbean sprout 200g				
			Skim Milk	250ml				
			Oil	15g			Oil	15g
DAY5	Steamed Yam	Yam 100g	Steamed Bread	140g (Flour 100g)	Watermelon	150g	Steamed Rice	165g (Rice 75g)
	Steamed Bread	75g (Flour 50g)	Saute Cabbage with Pork	Cabbage 200g	Skim Milk	250ml	Saute Spinage with Egg	Spinage 250g
	Egg	60g		Pork 75g				Egg 120g
	Skim Skim Milk	250ml	Saute Celery	Celery 200g				
			Skim Milk	250ml				
			Oil	15g			Oil	15g
DAY6	Bread	3 slice (Flour 75g)	Steamed Rice	220g (Rice 100g)	Strawberry	150g	Steamed Rice	165g (Rice 75g)
	Egg	60g	Saute Lettuce with Pork	Lettuce 200g	Skim Milk	250ml	Saute Cucumber with Egg	Cucumber 250g
	Skim Skim Milk	250ml		Pork 75g				Egg 120g
			Saute Cabbage	Cabbage 200g				
			Skim Milk	250ml				
			Oil	15g			Oil	15g
DAY7	Noodles	1 bowl (Flour 70g)	Steamed Rice	220g (Rice 100g)	Watermelon	150g	Steamed Rice	165g (Rice 75g)
		Cucumber 50g	Saute Eggplant with Pork	Eggplant 200g	Skim Milk	250ml	Saute Cabbage with Egg	Cabbage 250g
	Egg	60g		Pork 75g				Egg 120g
	Skim Skim Milk	250ml	Saute Celery	Celery 200g				
			Skim Milk	250ml				
			Oil	15g			Oil	15g

This dietary protocol can be adjusted according to actual conditions, and interchangeability is permitted within major food groups. Specifically, substitutions are allowed among different varieties within the same food group: different types of vegetables, livestock and poultry meat, staple foods, and fruits can be interchanged within their respective groups. If the intake of root and tuber vegetables is increased, the intake of staple foods shall be reduced following a 4:1 ratio (ratio of increased root and tuber vegetable intake to reduced staple food intake).

Supplementary Table S2 Effects of an LPEB Diet on Serum Biochemical Parameters: Per Protocol Analysis

**Time**	**Intervention group (n = 43)**	***P*-time**	**Control group (n = 36)**	***P*-time**	***P*-value**
**ALT**, U/L					
Day0	36.4 ± 21.5	0.009	41.9 ± 26.2	0.141	0.174
Day42	31.6 ± 17.8		37.8 ± 23.3		0.204
Change	-4.8 ± 11.3		-4.2 ± 16.9		0.597
**AST**, U/L					
Day0	26.2 ± 12.5	0.259	27.1 ± 10.6	0.124	0.932
Day42	24.8 ± 9.2		24.7 ± 8.6		0.639
Change	-1.5 ± 8.4		-2.4 ± 9.3		0.572
**TG**, mmol/L					
Day0	2.2 ± 1.0	＜ 0.001	1.8 ± 0.87	0.952	0.158
Day42	1.7 ± 0.8		1.8 ± 0.68		0.494
Change	-0.4 ± 0.5		0.0 ± 0.8		0.003
**TC**, mmol/L					
Day0	5.1 ± 1.0	0.044	4.9 ± 1.0	0.515	0.394
Day42	4.7 ± 0.7		4.8 ± 1.1		0.612
Change	-0.4 ± 0.8		-0.1 ± 0.8		0.205
**BG**, mmol/L					
Day0	5.7 ± 0.8	0.077	5.6 ± 1.1	0.646	0.513
Day42	5.5 ± 0.9		5.6 ± 0.9		0.577
Change	-0.2 ± 0.6		0.1 ± 0.8		0.471
**UA**, μmol/L					
Day0	463.2±75.3	<0.001	452.2±83.5	0.628	0.596
Day42	342.7±96.9		395.1±119.0		0.012
Change	-108.3(-241.1,25.1)		-26.5(-148.4,96.4)		0.048
**BUN**, mmol/L					
Day0	4.5 ± 1.6	0.021	5.4 ± 1.7	0.044	0.009
Day42	4.9 ± 1.2		5.1 ± 1.6		0.928

Change	0.5 ± 1.3		-0.4 ± 1.1		0.003
**CREA**, µmol/L					
Day0	84.5 ± 17.3	0.016	81.1 ± 17.5	0.415	0.201
Day42	80.6 ± 15.2		82.5 ± 18.0		0.787
Change	-3.8 ± 9.9		1.4 ± 10.4		0.009
**GFR**, mL/min/1.73 m^2					
Day0	96.1 ± 21.8	0.007	96.1 ± 22.2	0.679	0.566
Day42	101.8 ± 24.4		95.2 ± 21.5		0.415
Change	5.4 ± 12.4		-0.8 ± 11.6		0.020
**CREA-U**, mmol/L					
Day0	9.8 ± 8.2	0.018	11.1 ± 6.3	0.904	0.231
Day42	13.2 ± 7.5		11.2 ± 5.6		0.282
Change	3.3 ± 8.6		0.1 ± 6.4		0.037
**UA-U**, mmol/L					
Day0	1.8 ± 1.1	0.052	2.0 ± 1.1	0.937	0.214
Day42	2.2 ± 1.1		2.0 ± 1.1		0.638
Change	0.4 ± 1.3		0.0 ± 1.1		0.109
**FEUA**, %					
Day0	4.0 ± 2.2	0.088	4.3 ± 1.9	0.490	0.952
Day42	4.9 ± 2.7		4.0 ± 1.4		0.012
Change	0.9 ± 1.8		-0.3 ± 1.5		0.003
**pH-U**					
Day0	5.8 ± 0.6	0.874	5.9 ± 0.7	0.497	0.339
Day42	5.8 ± 0.6		5.8 ± 0.6		0.522
Change	0.0 ± 0.5		0.1 ± 1.0		0.522

P-values for within-group changes (pre- vs. post-intervention) were calculated using paired t-tests. *P*-values for between-group differences were derived from statistical models adjusted for potential confounding factors. Abbreviation: ALT, alanine aminotransferase; AST, aspartate aminotransferase; TC, total cholesterol; TG, triglycerides; BG, blood glucose; UA, uric acid; BUN, blood urea nitrogen; CREA, creatinine; GFR, glomerular filtration rate; CREA-U, urinary creatinine; UA-U, urinary uric acid; FEUA, fraction excretion of uric acid, calculated as (urinary uric acid × serum creatinine) / (serum uric acid × urinary creatinine) × 100%; pH-U, urine pH
